# Code and data on the categorization of soft-drink bottles using image silhouettes

**DOI:** 10.1016/j.dib.2018.12.068

**Published:** 2018-12-24

**Authors:** Ana M. Arboleda, Carlos Arce-Lopera

**Affiliations:** Universidad Icesi, Cali, Colombia

## Abstract

This data article includes the visual stimuli used to test the categorization of a set of soft drink bottle silhouettes. Additionally, subjects’ perceptual categorization was associated with each visual stimuli. The silhouette of the soft drink bottles was characterized by calculating the most common object shape measurements such as width, height and area and combining them with more complex and specific quantitative shape measurements such as the principal moment statistics. Finally, this data article includes the code for extracting these shape characteristics from image silhouettes. For interpretation and discussion, please see the original article entitled “Quantitative analysis of product categorization in soft drinks using bottle silhouettes” (Arboled and Arce-Lopera, 2015) [1].

**Specifications table**

**Table:** 

Subject area	*Psychology*
More specific subject area	*Consumer Perception*
Type of data	*MATLAB code, JPEG images,.CSV file.*
How data was acquired	*Digital Optical Camera, Canon EOS7D for the images. Code for the shape characteristics. And a psychophysical experiment for the consumer perception results.*
Data format	*Filtered*
Experimental factors	*Images were filtered to extract the silhouettes.*
Experimental features	*Subjects were presented with the bottle silhouette images and asked to categorize them into one of seven possible options; namely, soda, fruit juice, water, tea, sports drink, flavored water and malt.*
Data source location	*Cali, Colombia*
Data accessibility	*The data are attached to this article.*
Related research article	*Arboleda A.M., Arce-Lopera C.A. Quantitative analysis of product categorization in soft drinks using bottle silhouettes. Food Quality and Preference. 2015; 45: 1–10.*

**Value of the data**

• The stimuli can be used in the development of further experiments and to test different types of consumer perceptions. The visual stimuli can be exploited in different research areas, such as psychology, sensory science, and marketing. For example, in psychology, an experiment could test subjects’ perceived quality or price assumptions. For sensory science, psychophysical experiments could explain the influence of a bottle shape on taste or nutrient estimates. Finally, for marketing, the visual stimuli could be used to describe the relationship between branding and product shape characteristics.
• The data collected from the bottle silhouettes are comparable with other international silhouette data from images of different products, such as the CalTech 101 Silhouettes Data Set [2].
• The experimental results of the categorization can be used as a guide for the design of soft drink bottles.
• Researchers can try out the code to extract the shape information from their own product silhouettes.

## 1 Data

The data consist of four main components:

1) The stimuli, a zip compressed file with 52 jpg silhouette images (see Stimuli.zip file).
2) The code, a MATLAB code that extracts shape measures from silhouette images (see ShapeMeasure.m).
3) The extracted shape data, a csv file with the results of the shape measurements from the bottle silhouettes (see BottleShapeInfo.csv).
4) The results of the psychophysical experiment, a csv file with the results from the experiments performed in [1] (see ExperimentalResults.csv).

## 2 Experimental design, materials, and methods

### 2.1 Stimuli

**Fig. 1:**
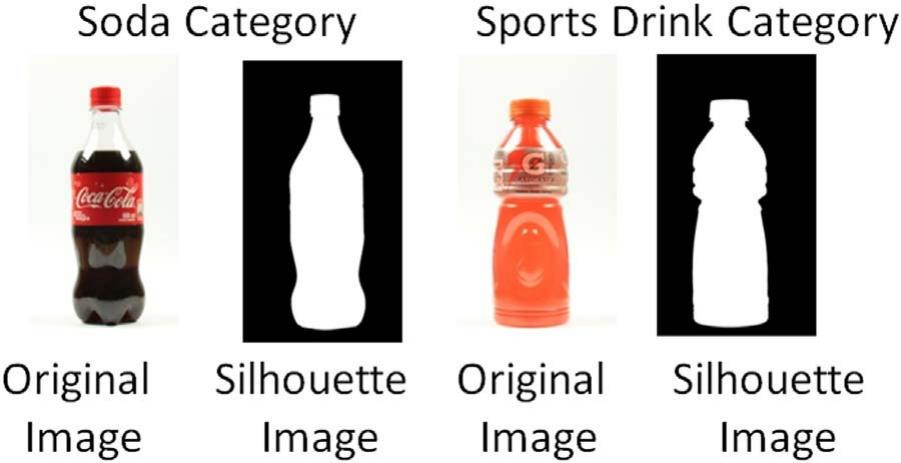
Original and silhouette example images of the soda and sport drink category.

The front side of 52 different personal-sized bottles (less than 600 ml) were photographed using a digital camera (Canon EOSD7) in a light-controlled environment. Then, from each high-resolution digital image, the bottle silhouette was extracted using image processing techniques. Only the information of the bounding box circumscribed to the silhouette (see Fig. 1) was stored as visual stimuli (see Stimuli.zip file). The content of the bottles belonged to one of seven soft-drink categories; namely, soda (16 different shapes), water (8 different shapes), fruit juice (11 different shapes), tea (7 different shapes), sports drink (3 different shapes), flavored water (3 different shapes) or malt drink (4 different shapes). These soft-drink bottles were purchased in Cali, Colombia and some of them, particularly the malt drinks, are specific to the Colombian market.

## 3 Code

**Fig. 2:**
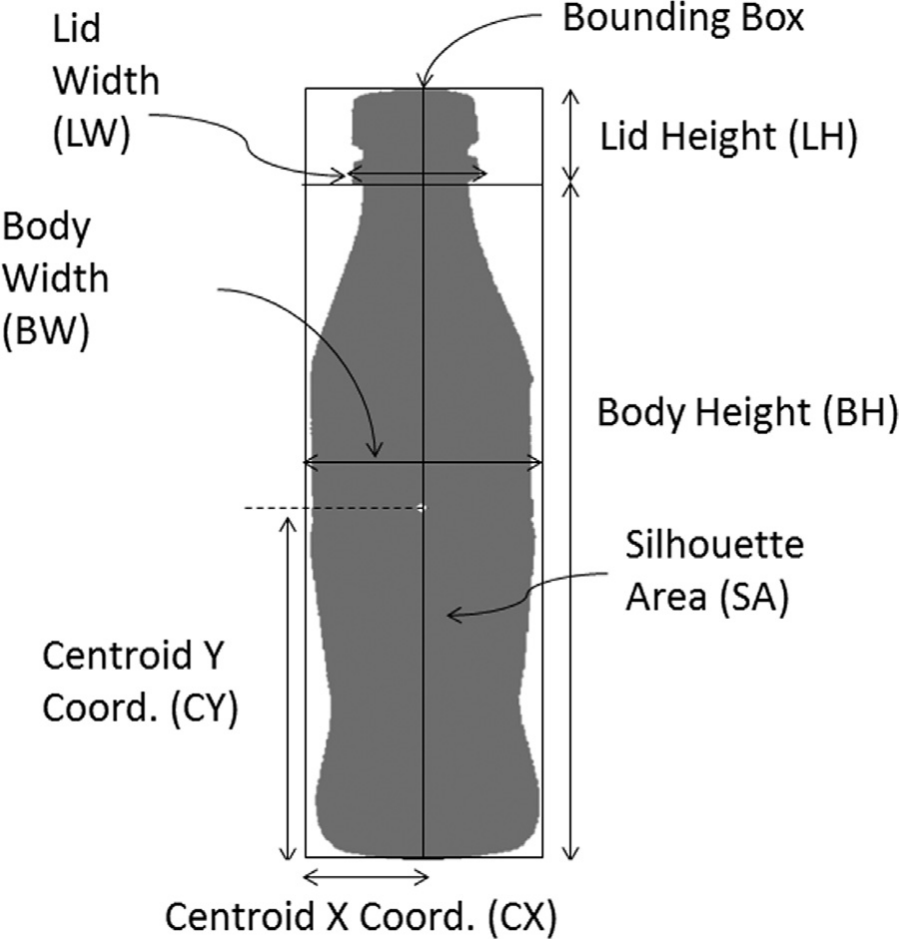
Global measures of the silhouettes.

A MATLAB code was implemented to take a silhouette image as an input and measure physical properties that can be related to their shape. All silhouette images in JPEG format in a folder are loaded by the computer program. Then, global measures from the bounding box are taken; namely, the Centroid X coordinate (CX), Centroid Y coordinate (CY), Body Height (BH), Body Width (BW) and Silhouette Area (SA). Subsequently, the algorithm searches for the smallest diameter to separate the lid from the body of the bottle. Finally, the Lid Height (LH) and Lid Width (LW) were measured. Fig. 2 shows a schematic view of these general shape measurements.

**Fig. 3:**
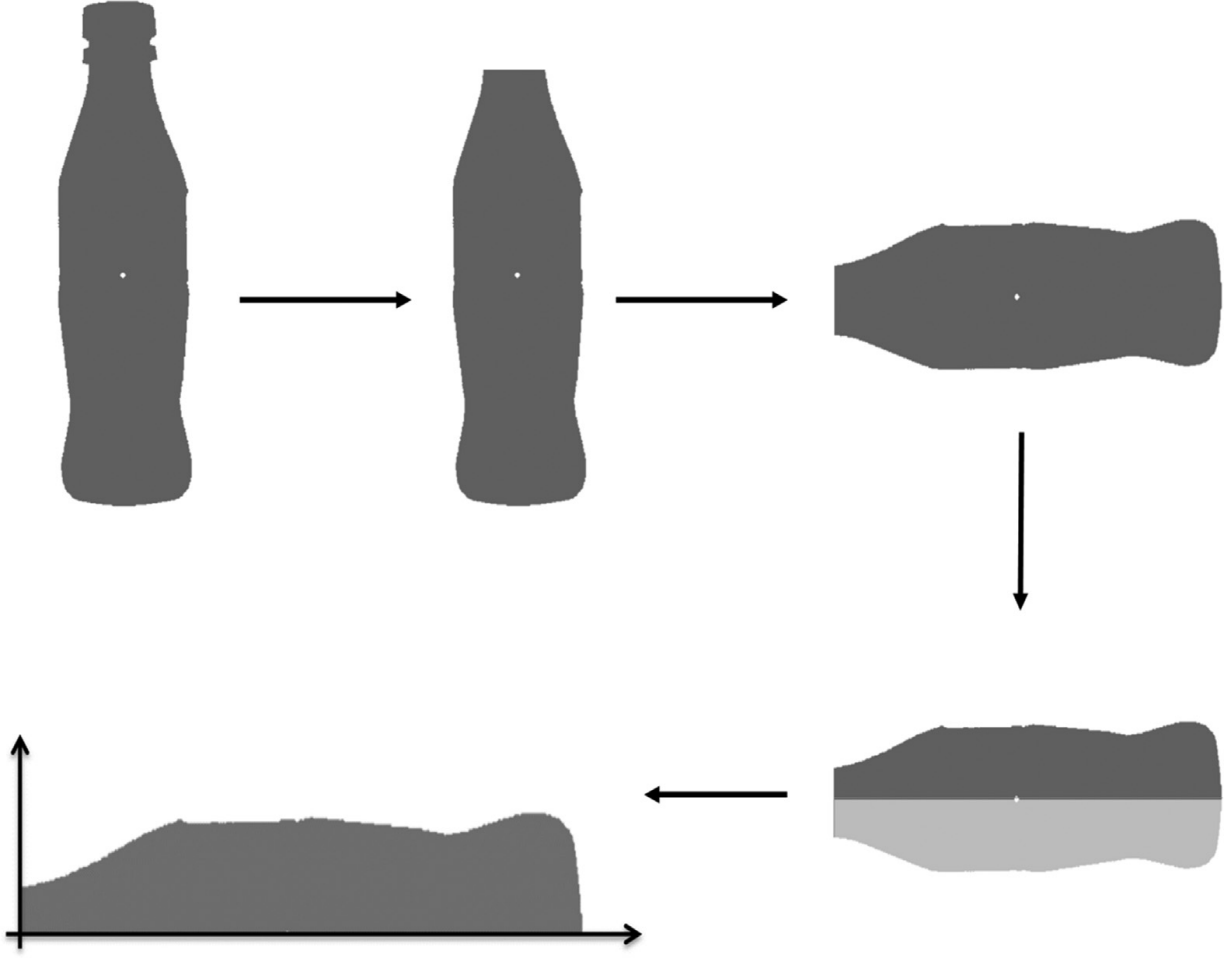
Procedure to transform bottle silhouettes into distributions. Only the right side of the bottle body is considered.

Additionally, to measure physical characteristics that were directly related to shape of the body, we transformed the side of the bottle to a distribution and calculated four statistical features: Body Mean (BM), Body Variance (BV), Body Skewness (BS), and Body Kurtosis (BK). To achieve this shape encoding, only the Body of the silhouette was considered. The body image was rotated 90 degrees so that the lid was on the left side and the bottom on the right. Then, the silhouette was transformed to a distribution using only the top information from the shape (see Fig. 3). The code was written in the MATLAB programming language (see ShapeMeasure.m file).

## 4 Shape data results

The MATLAB code generated the measurement data of the bottle silhouettes in CSV format. The CSV file is organized as follows: The first column was the name of the file, the following four columns were the shape statistics (BM, BV, BS, BK). Then, the following columns were the seven global characteristics (CX, CY, BH, BW, SA, LH, LW). This resulted in a table with twelve columns. Each row in the table represents one silhouette image (see BottleShapeInfo.csv file). Values inside the table were based on the pixels of the original image. We calibrated the distance from the camera to the bottle stimuli; this allowed to transform the variables’ units from pixels to centimeters. Distance calibration need only two values, the distance from the camera to the object and the size of a standard object. Normally, to calibrate the camera a planar target image, namely a black and white checkerboard pattern, is used.

## 5 Psychophysical experiment results

26 individuals observed the stimuli displayed on a computer screen. While looking to the images, subjects were asked to assign the bottle silhouettes to one of the following categories: soda, fruit juice, tea, water, sports drink, flavored water, and malt drink. There were 156 trials (52 images × 3 repetitions) for each subject. For further details, please refer to [1]. The data results are stored in CSV format. Therefore, the experimental results consisted of a table with 7 columns and 4056 rows (26 individuals x 156 trials). The columns were the following: Subject ID, Age of the subject, biological sex (male or female), real category of the stimuli, name of the image file, perceived category of the silhouette and number of trial (see ExperimentalResults.csv file).
