# Supplementary figures and images for: Code and data on the categorization of soft-drink bottles using image silhouettes
Source: Data Brief. 2018 Dec 24;22:590–4. doi: 10.1016/j.dib.2018.12.068 (PMC6327070; doi:10.1016/j.dib.2018.12.068)

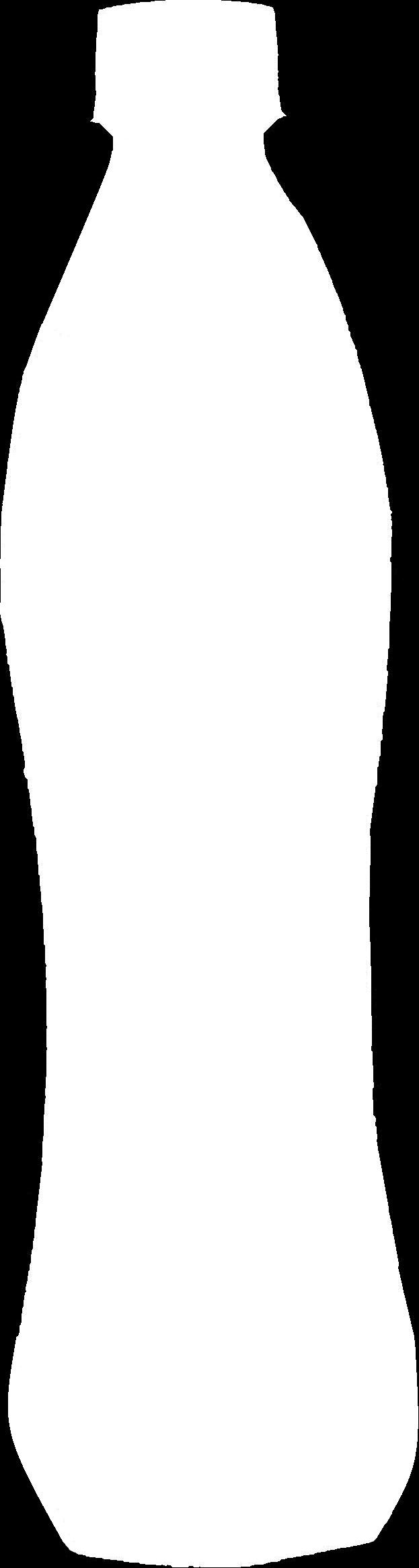

Supplement: Supplementary file 4 — Supplementary material [file mmc4.zip › Stimuli/FlavWater1.JPG]

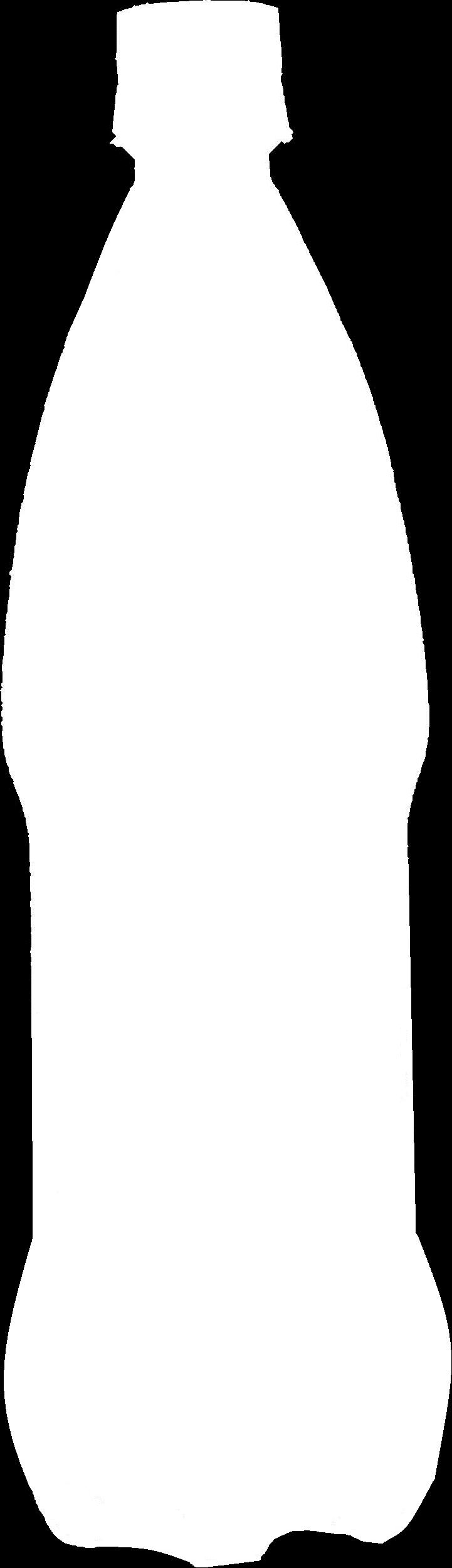

Supplement: Supplementary file 4 — Supplementary material [file mmc4.zip › Stimuli/FlavWater2.JPG]

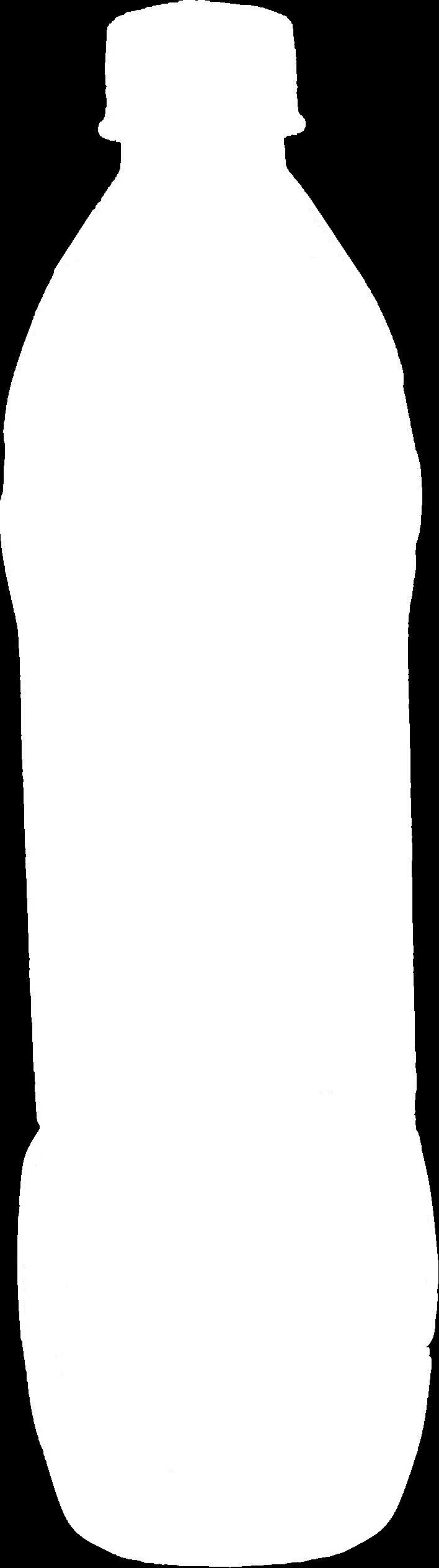

Supplement: Supplementary file 4 — Supplementary material [file mmc4.zip › Stimuli/FlavWater3.JPG]

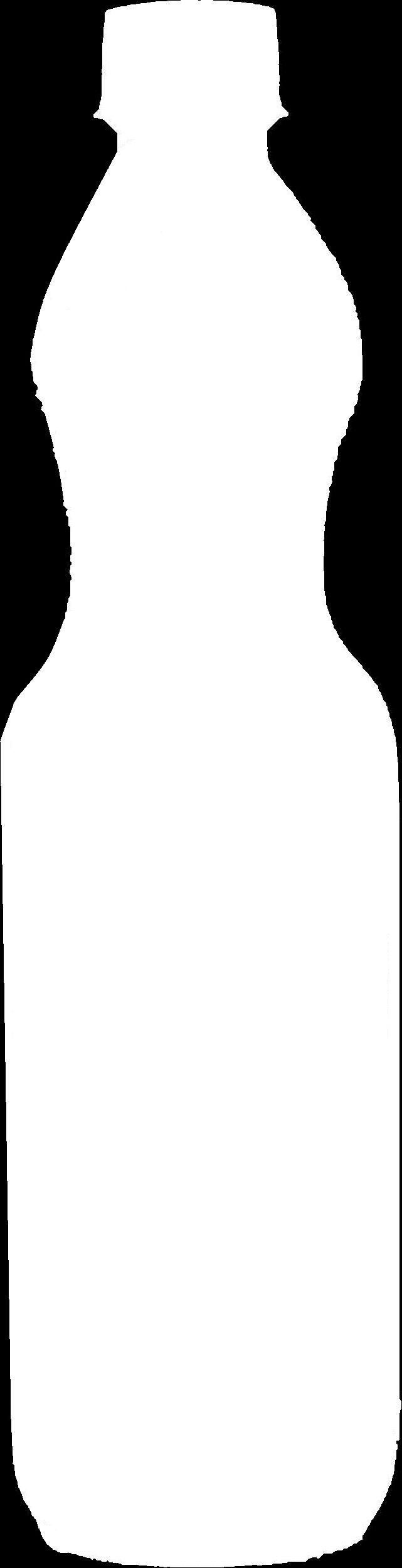

Supplement: Supplementary file 4 — Supplementary material [file mmc4.zip › Stimuli/FruitJuices1.JPG]

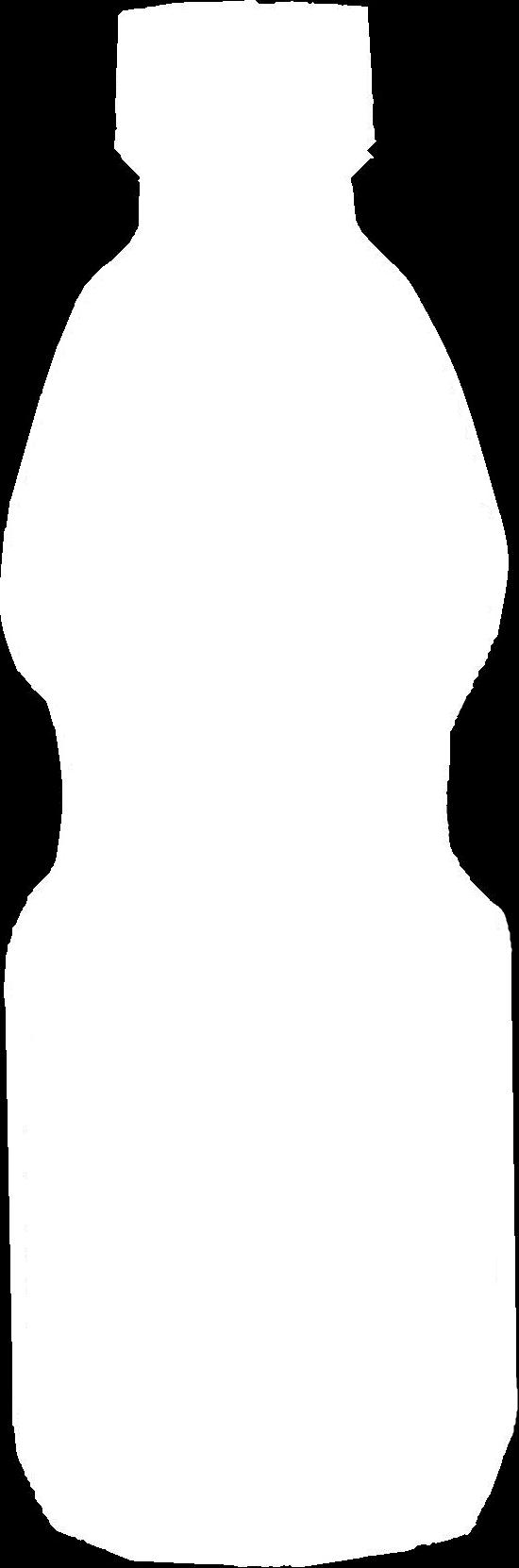

Supplement: Supplementary file 4 — Supplementary material [file mmc4.zip › Stimuli/FruitJuices10.JPG]

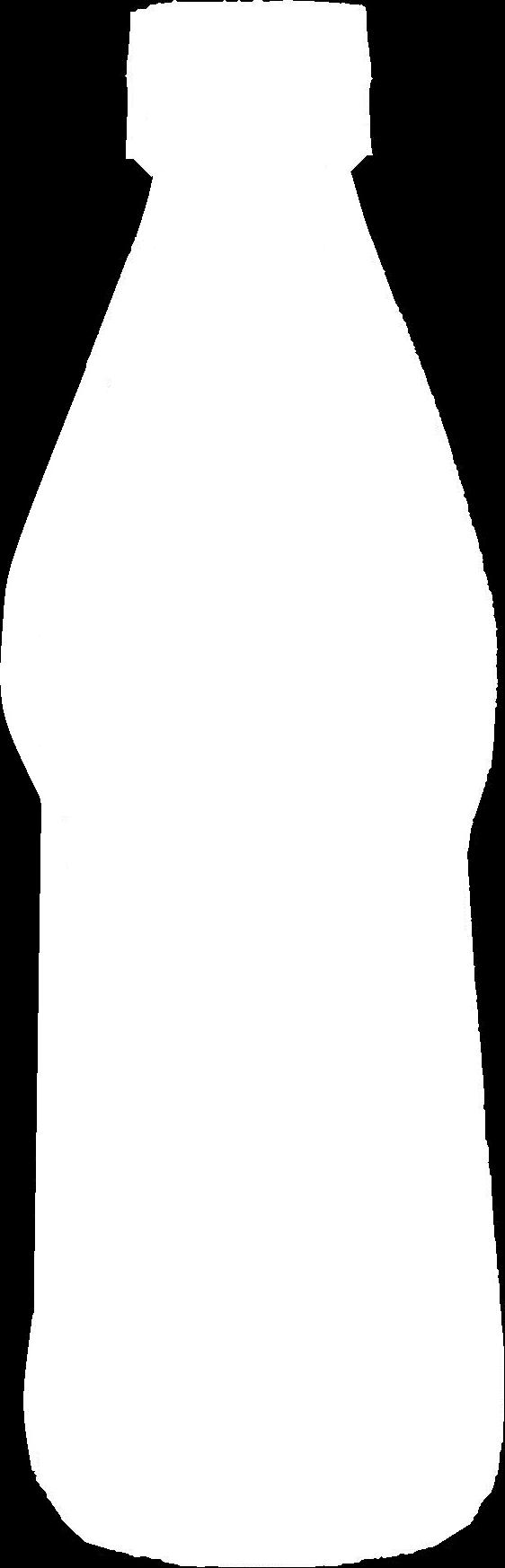

Supplement: Supplementary file 4 — Supplementary material [file mmc4.zip › Stimuli/FruitJuices11.JPG]

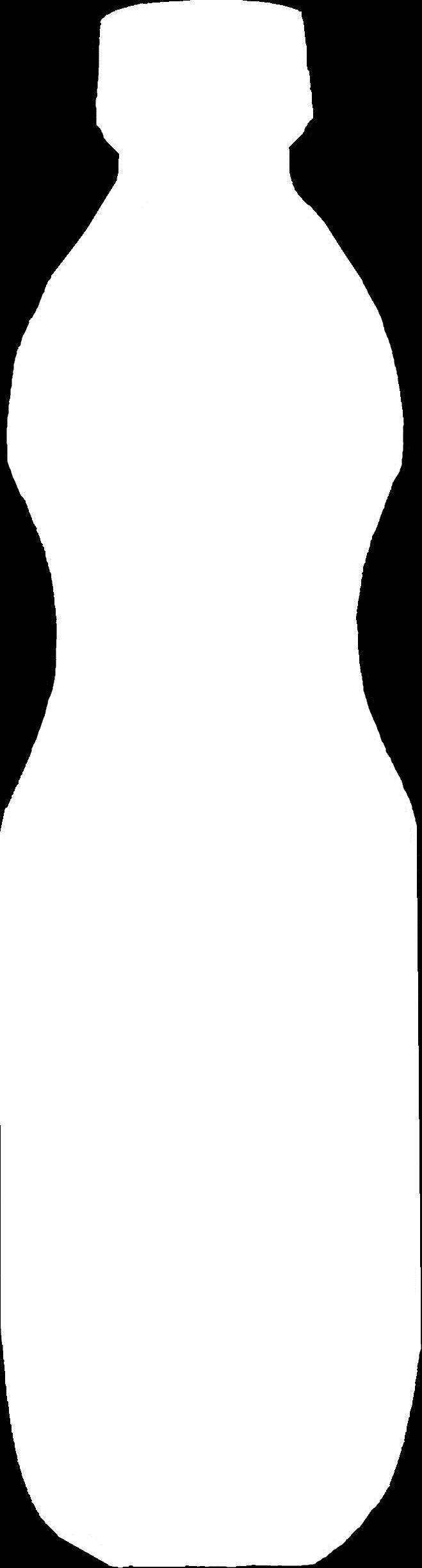

Supplement: Supplementary file 4 — Supplementary material [file mmc4.zip › Stimuli/FruitJuices2.JPG]

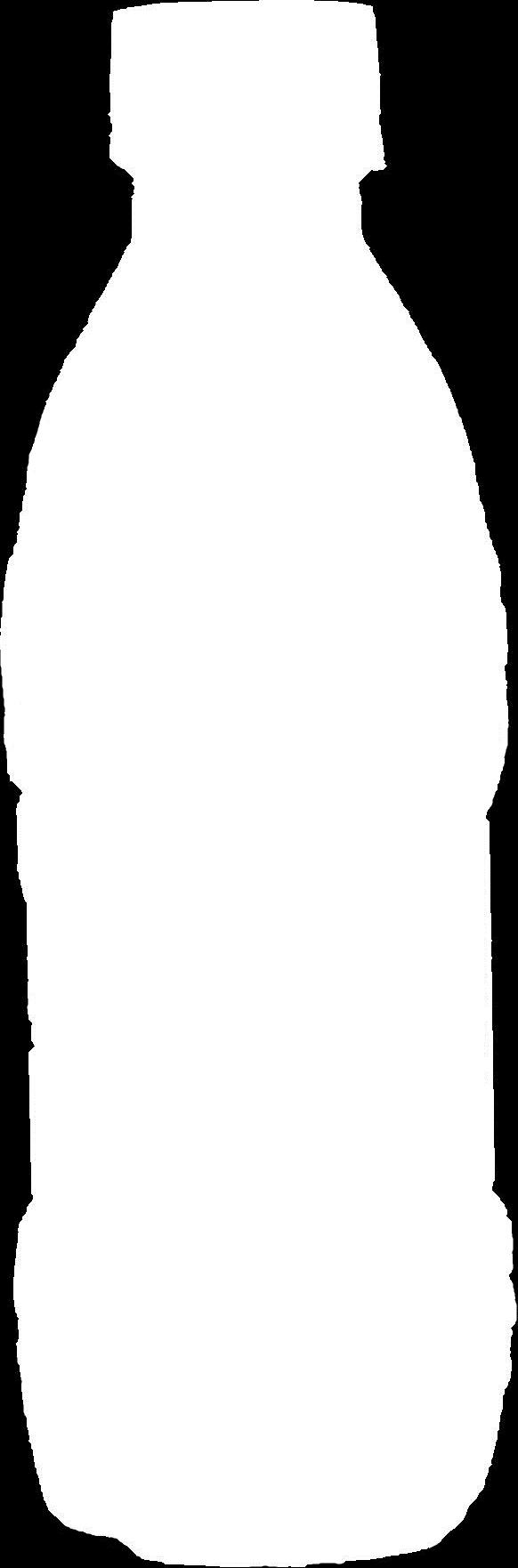

Supplement: Supplementary file 4 — Supplementary material [file mmc4.zip › Stimuli/FruitJuices3.JPG]

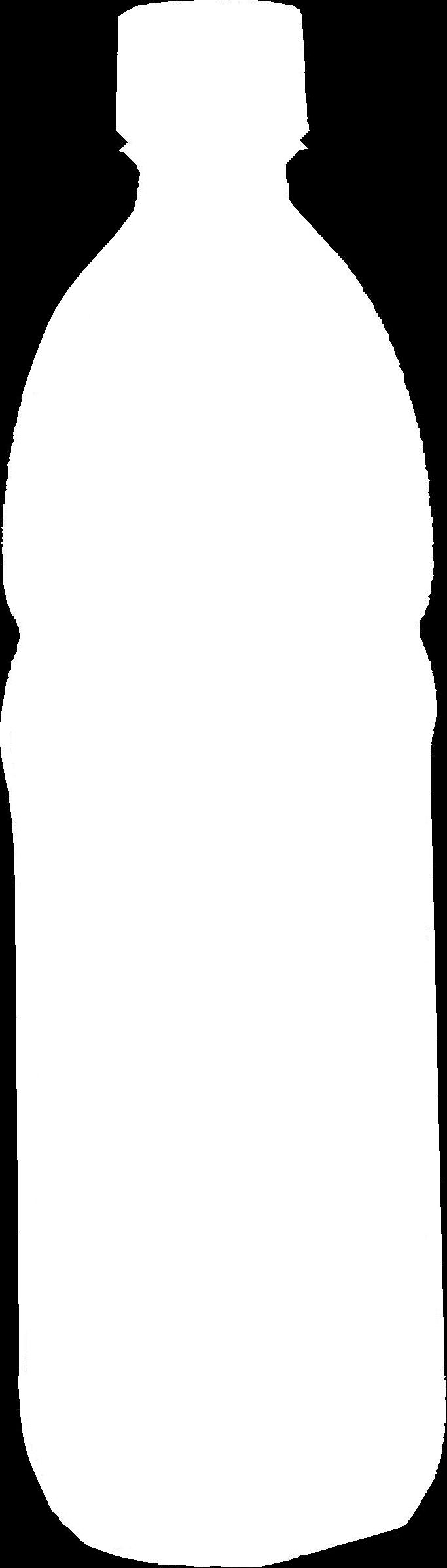

Supplement: Supplementary file 4 — Supplementary material [file mmc4.zip › Stimuli/FruitJuices4.JPG]

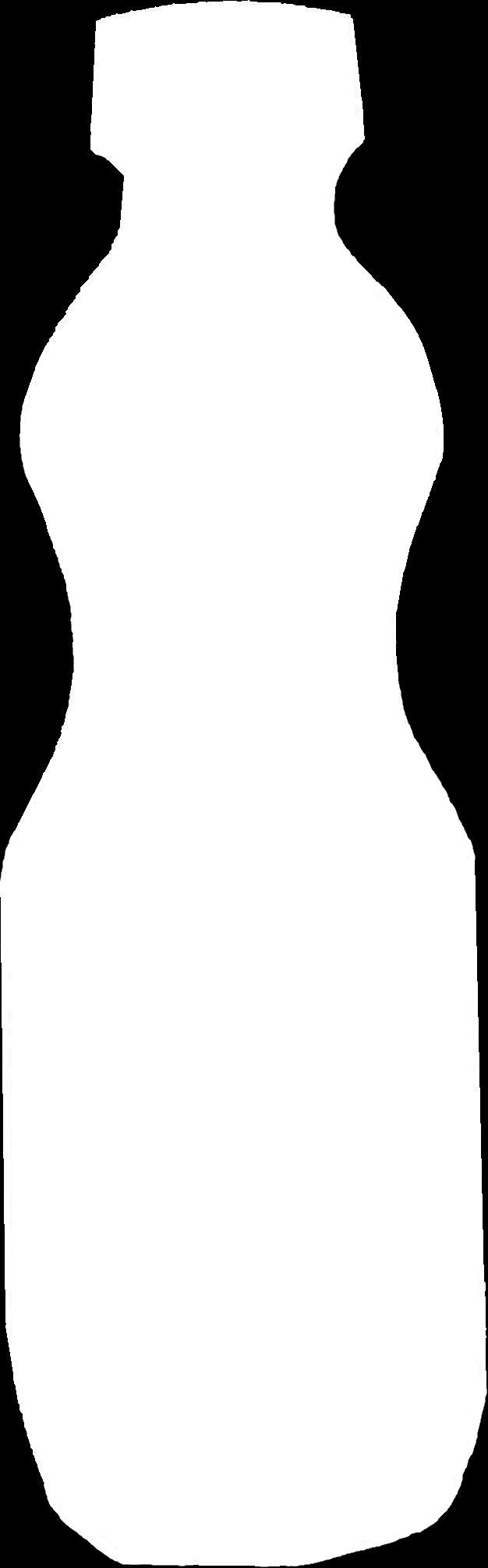

Supplement: Supplementary file 4 — Supplementary material [file mmc4.zip › Stimuli/FruitJuices5.JPG]

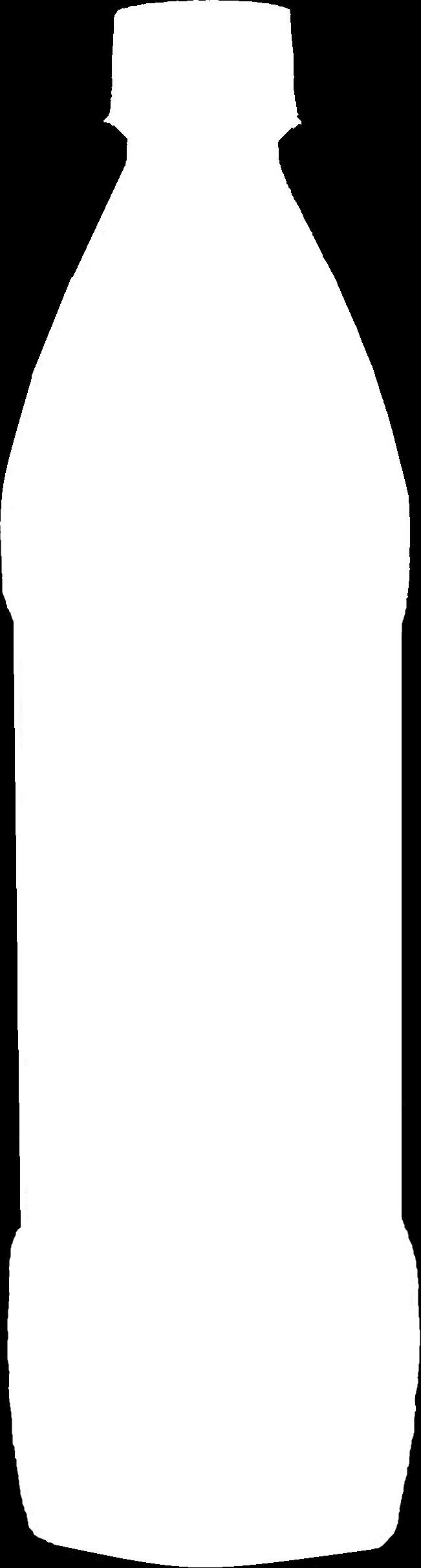

Supplement: Supplementary file 4 — Supplementary material [file mmc4.zip › Stimuli/FruitJuices6.JPG]

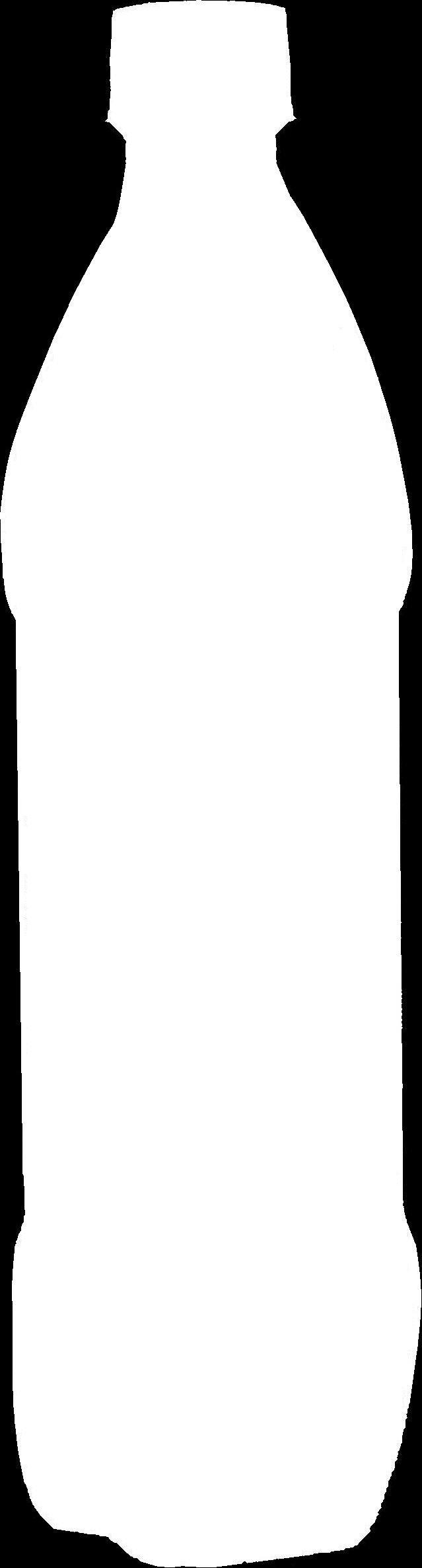

Supplement: Supplementary file 4 — Supplementary material [file mmc4.zip › Stimuli/FruitJuices7.JPG]

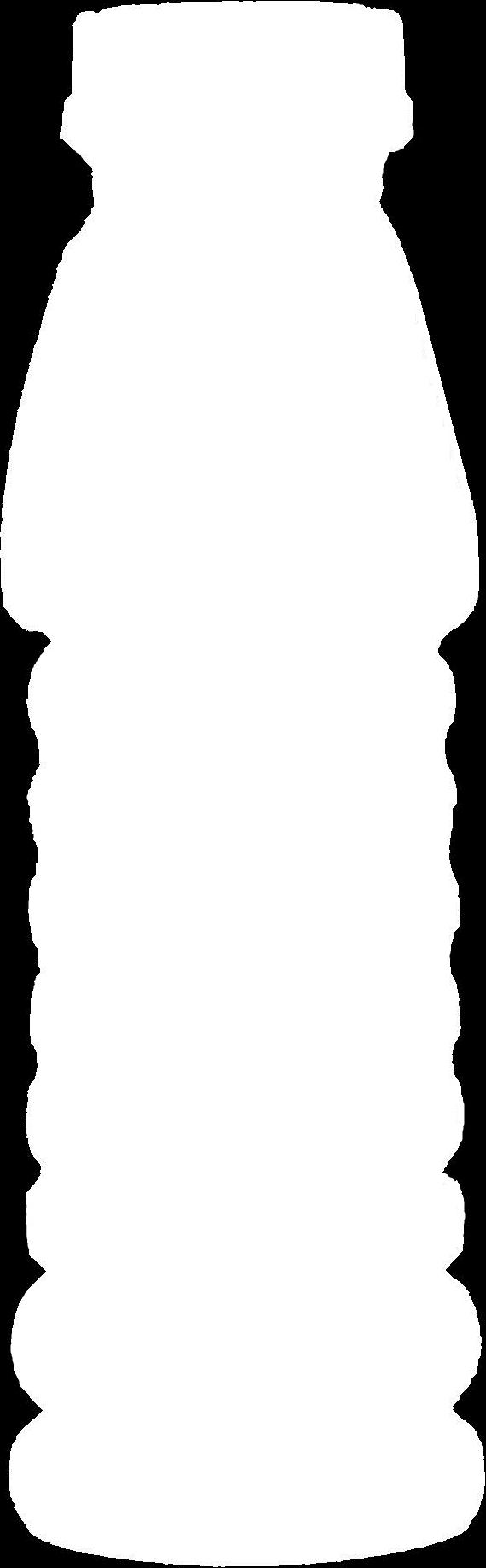

Supplement: Supplementary file 4 — Supplementary material [file mmc4.zip › Stimuli/FruitJuices8.JPG]

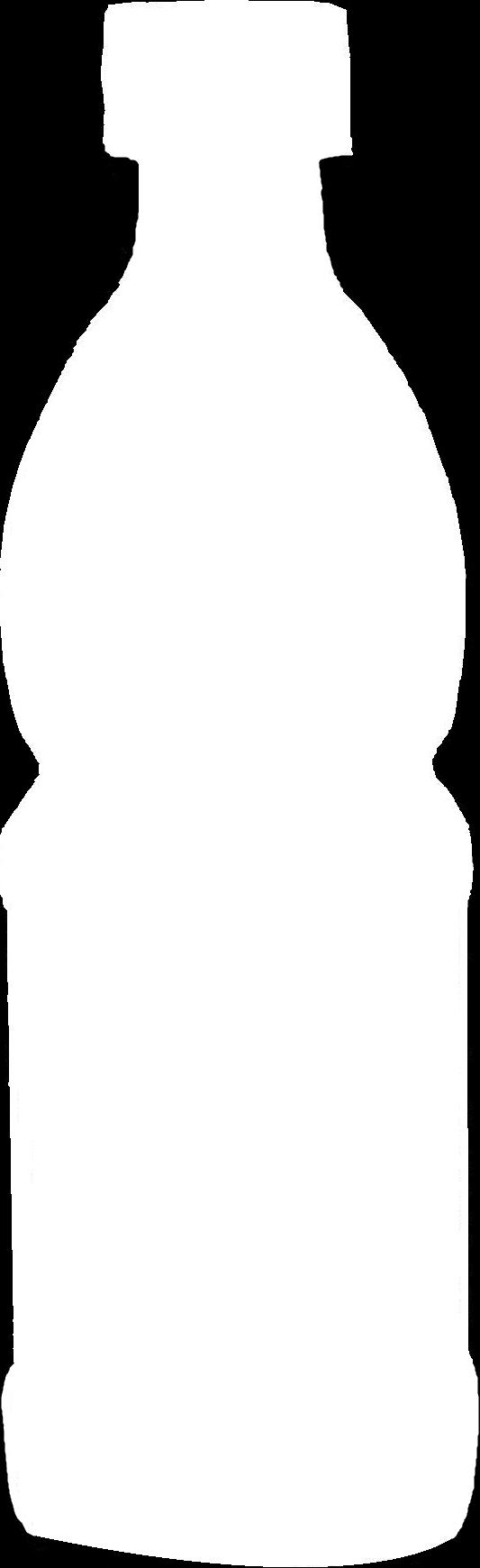

Supplement: Supplementary file 4 — Supplementary material [file mmc4.zip › Stimuli/FruitJuices9.JPG]

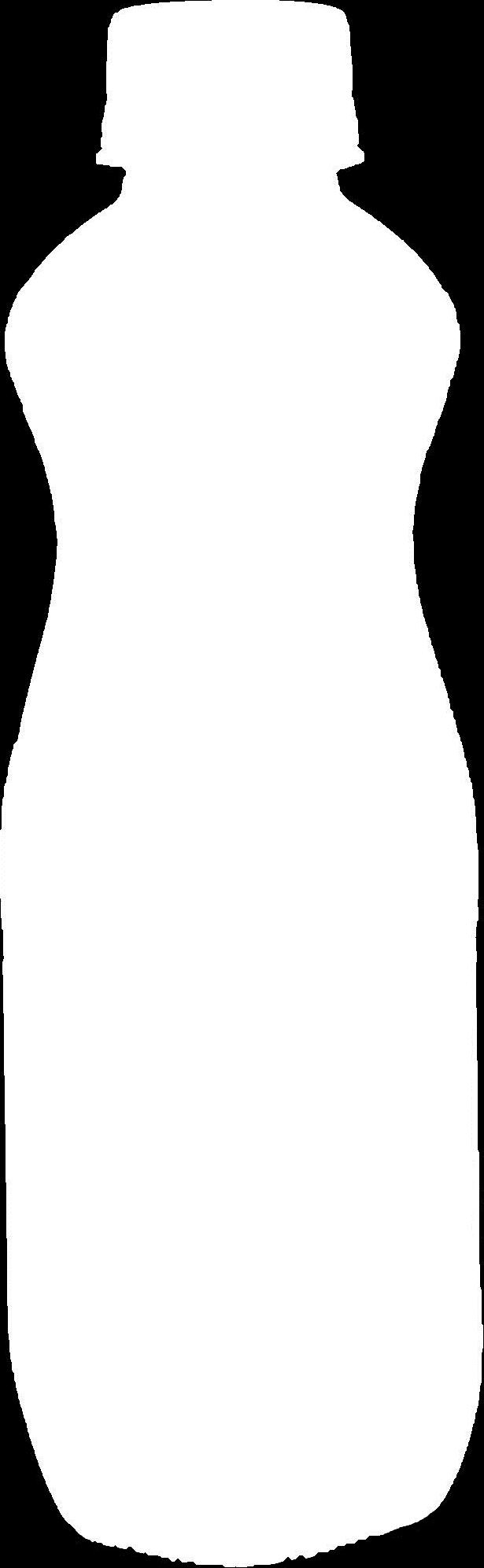

Supplement: Supplementary file 4 — Supplementary material [file mmc4.zip › Stimuli/MaltDrink1.JPG]

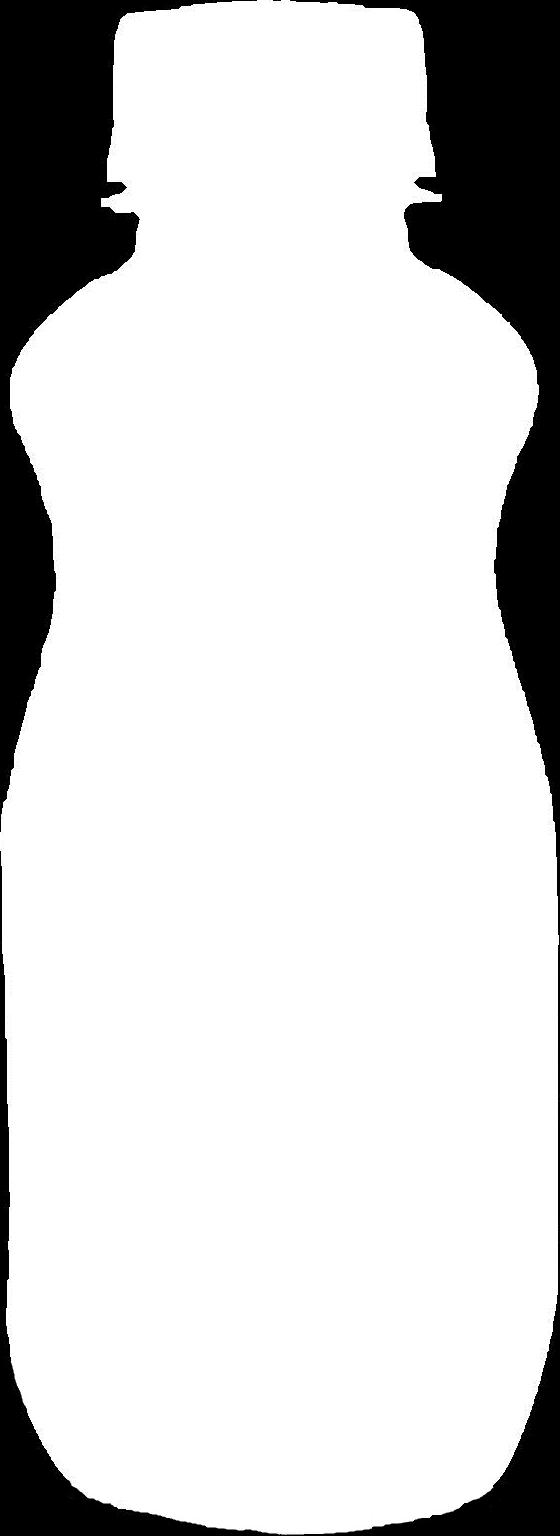

Supplement: Supplementary file 4 — Supplementary material [file mmc4.zip › Stimuli/MaltDrink2.JPG]

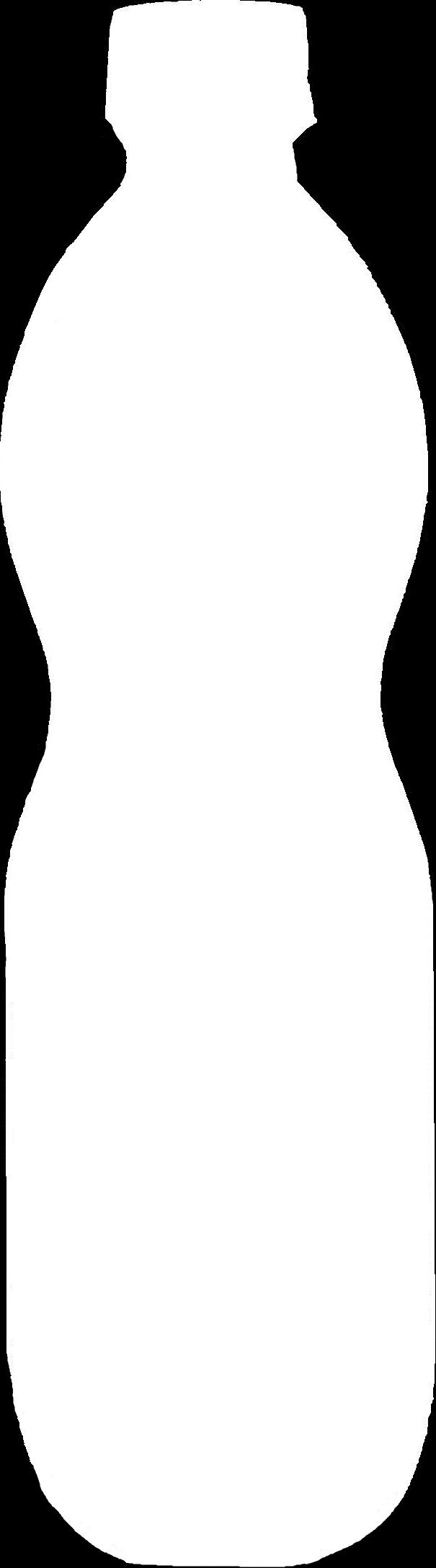

Supplement: Supplementary file 4 — Supplementary material [file mmc4.zip › Stimuli/MaltDrink3.JPG]

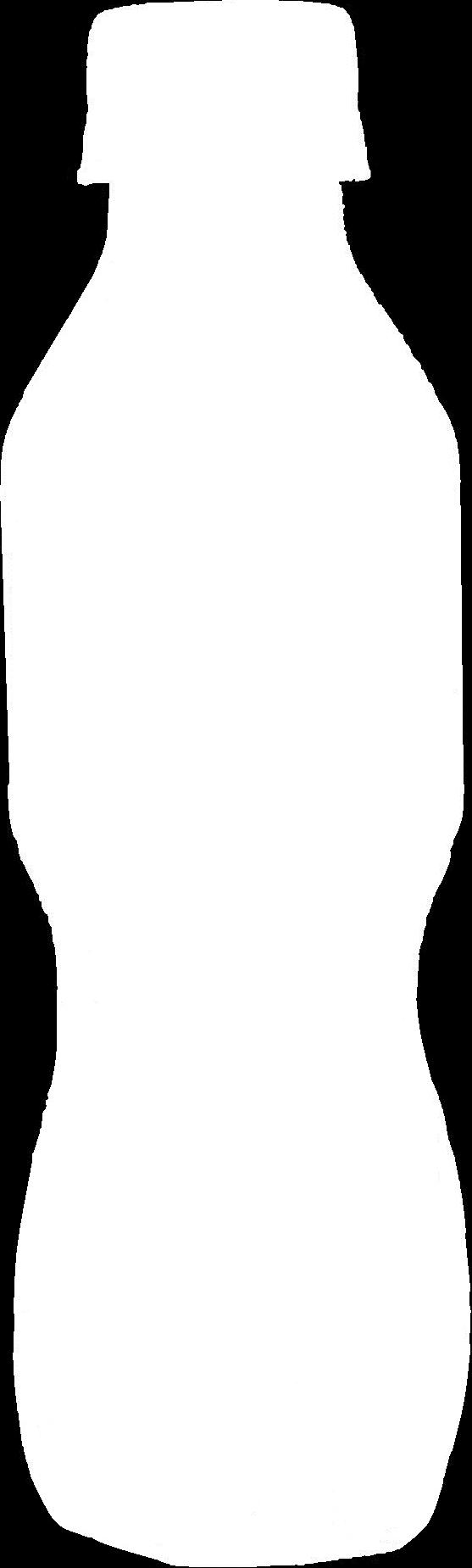

Supplement: Supplementary file 4 — Supplementary material [file mmc4.zip › Stimuli/MaltDrink4.JPG]

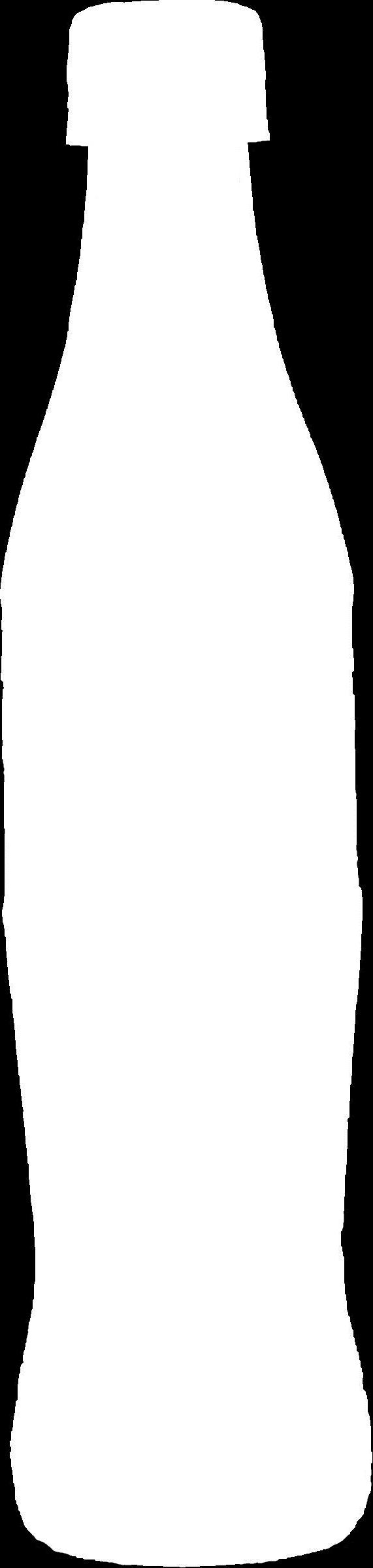

Supplement: Supplementary file 4 — Supplementary material [file mmc4.zip › Stimuli/Soda1.JPG]

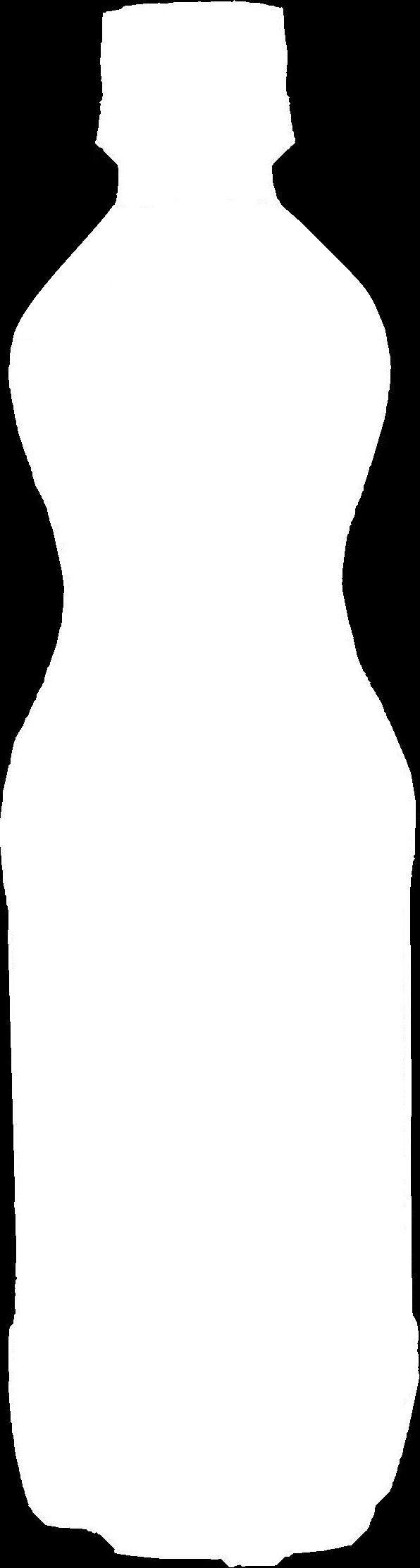

Supplement: Supplementary file 4 — Supplementary material [file mmc4.zip › Stimuli/Soda10.JPG]

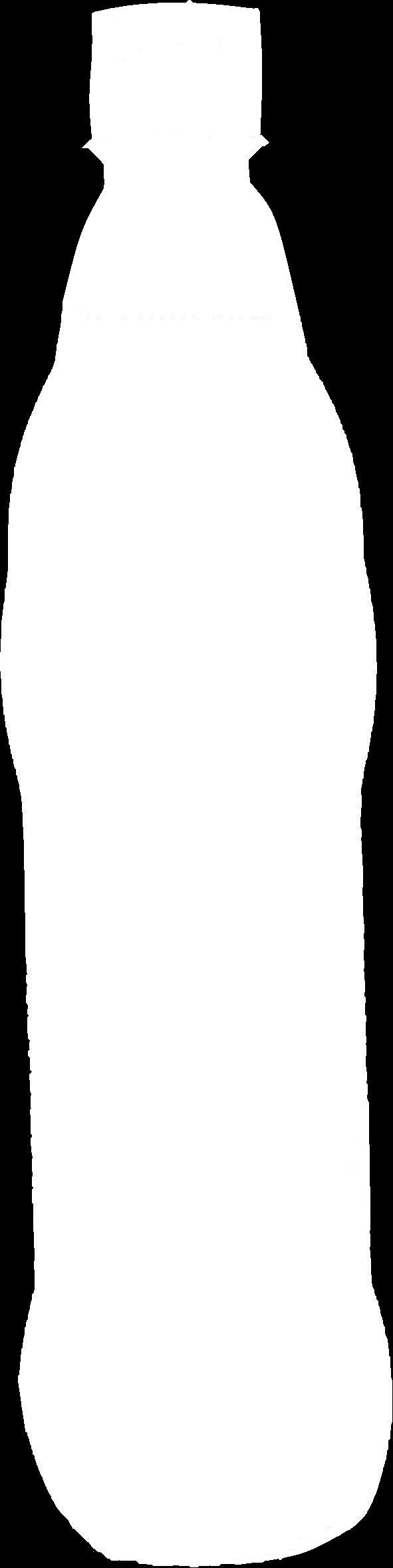

Supplement: Supplementary file 4 — Supplementary material [file mmc4.zip › Stimuli/Soda11.JPG]

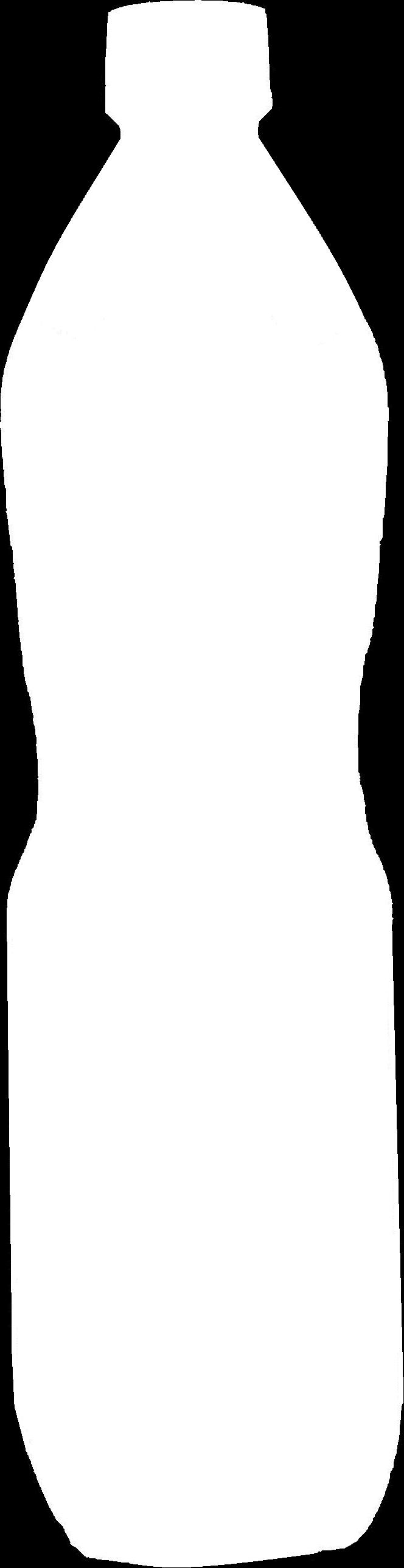

Supplement: Supplementary file 4 — Supplementary material [file mmc4.zip › Stimuli/Soda12.JPG]

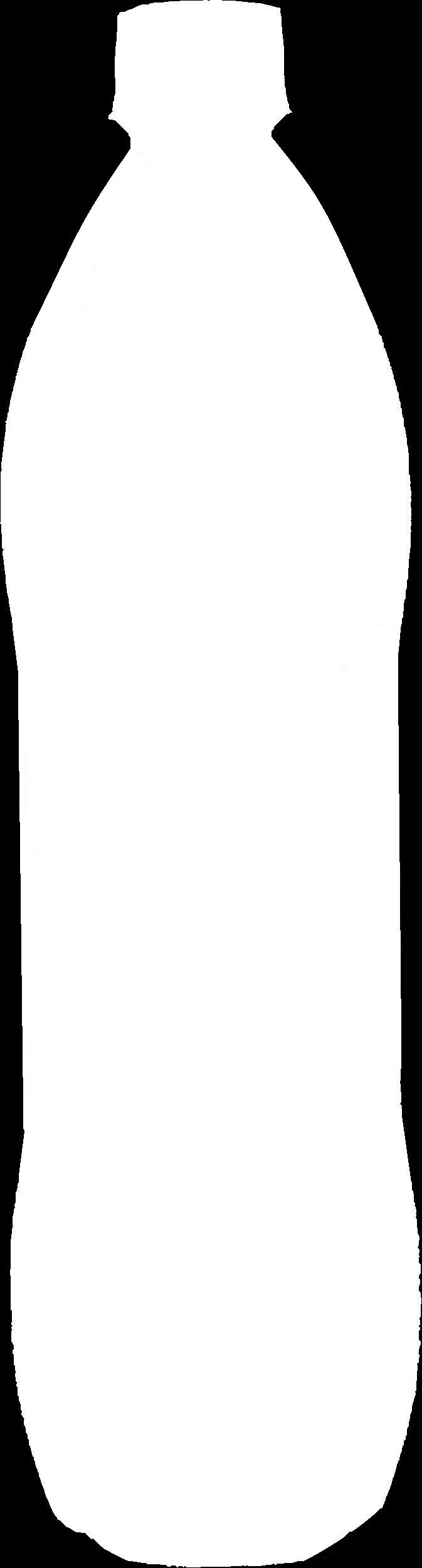

Supplement: Supplementary file 4 — Supplementary material [file mmc4.zip › Stimuli/Soda13.JPG]

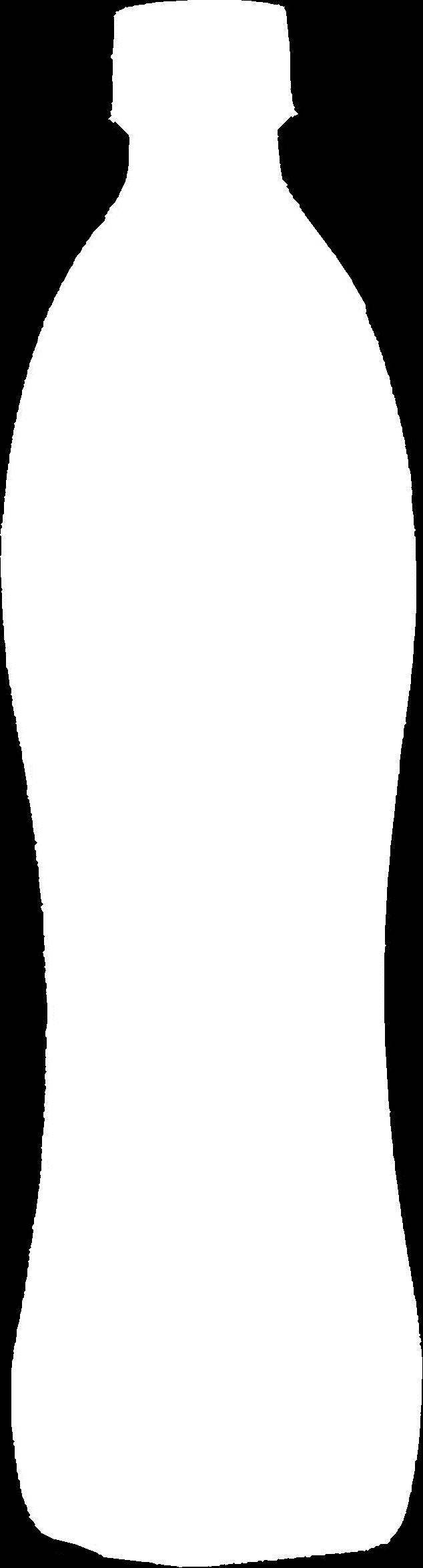

Supplement: Supplementary file 4 — Supplementary material [file mmc4.zip › Stimuli/Soda14.JPG]

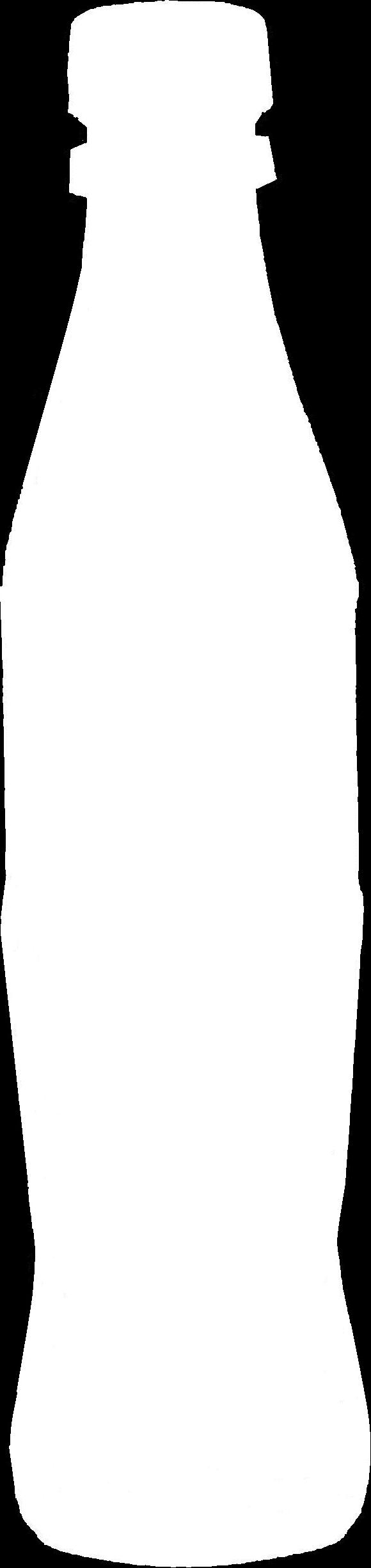

Supplement: Supplementary file 4 — Supplementary material [file mmc4.zip › Stimuli/Soda15.JPG]

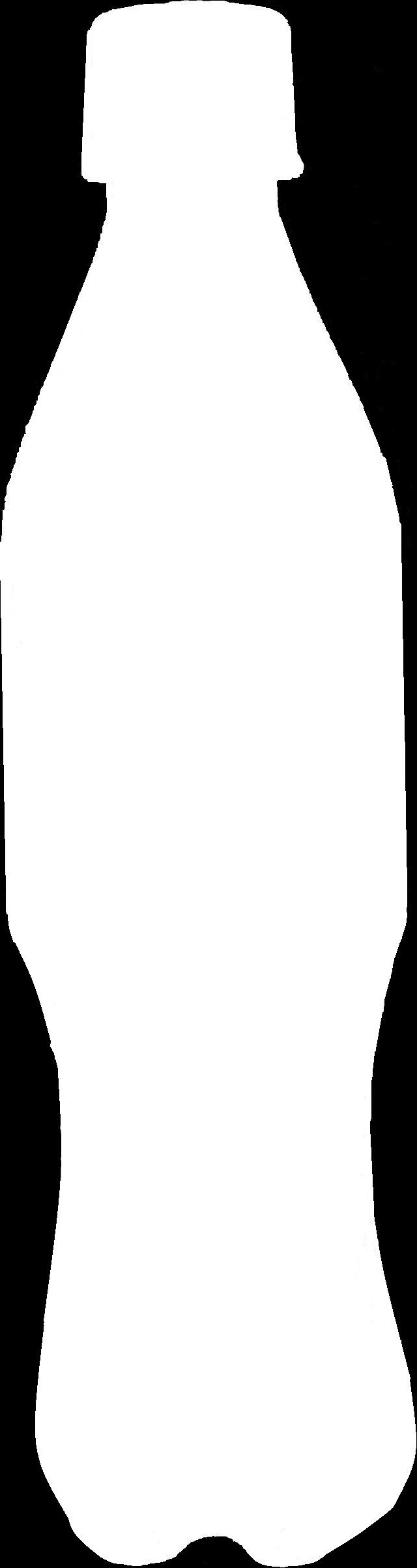

Supplement: Supplementary file 4 — Supplementary material [file mmc4.zip › Stimuli/Soda16.JPG]

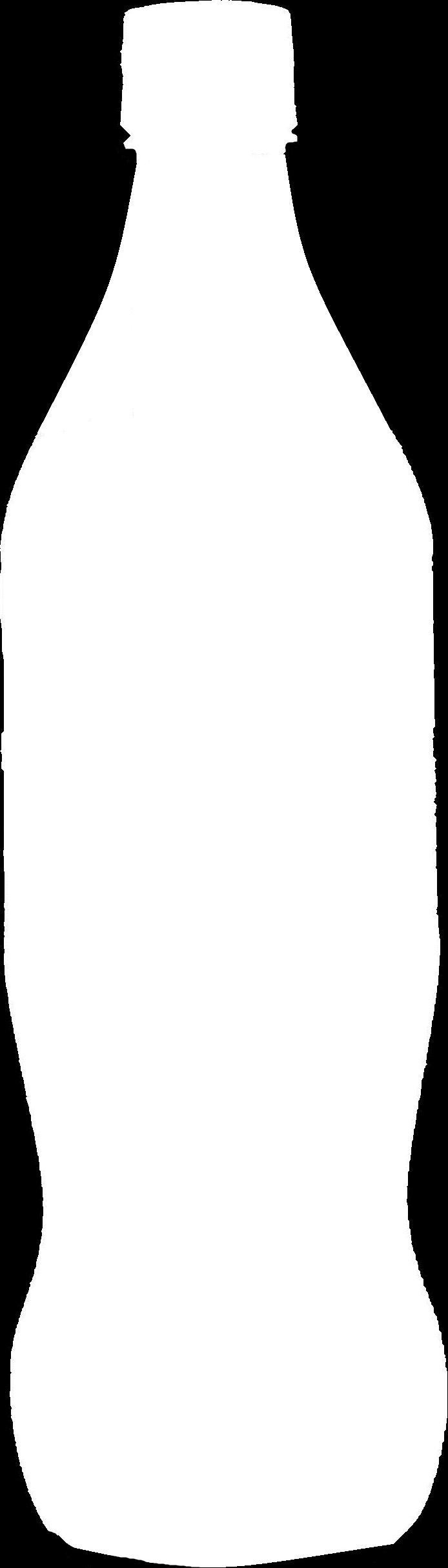

Supplement: Supplementary file 4 — Supplementary material [file mmc4.zip › Stimuli/Soda2.JPG]

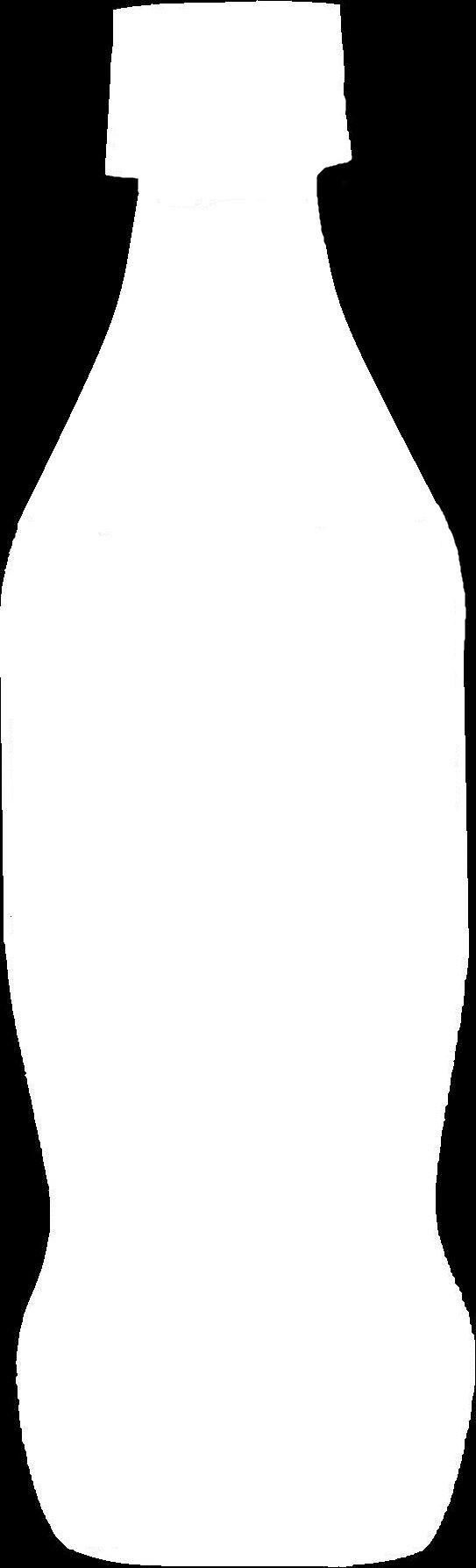

Supplement: Supplementary file 4 — Supplementary material [file mmc4.zip › Stimuli/Soda3.JPG]

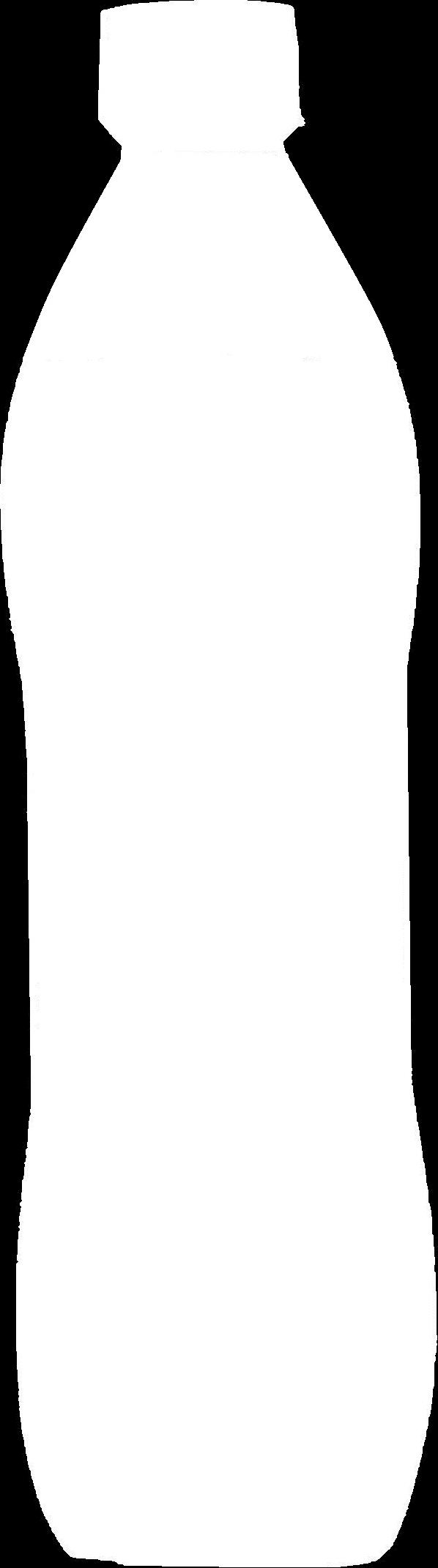

Supplement: Supplementary file 4 — Supplementary material [file mmc4.zip › Stimuli/Soda4.JPG]

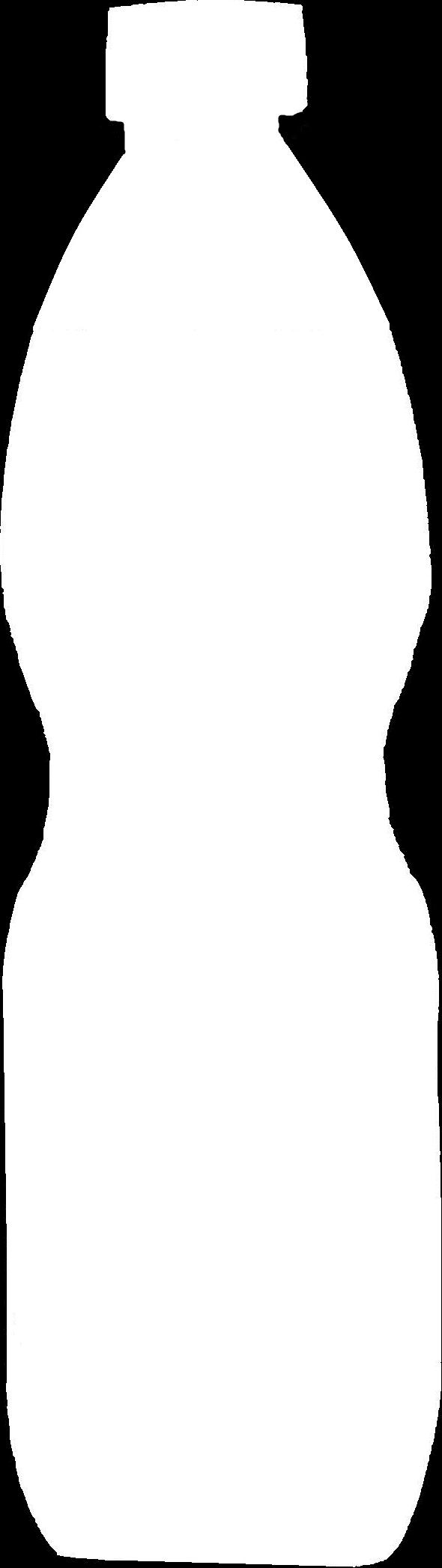

Supplement: Supplementary file 4 — Supplementary material [file mmc4.zip › Stimuli/Soda5.JPG]

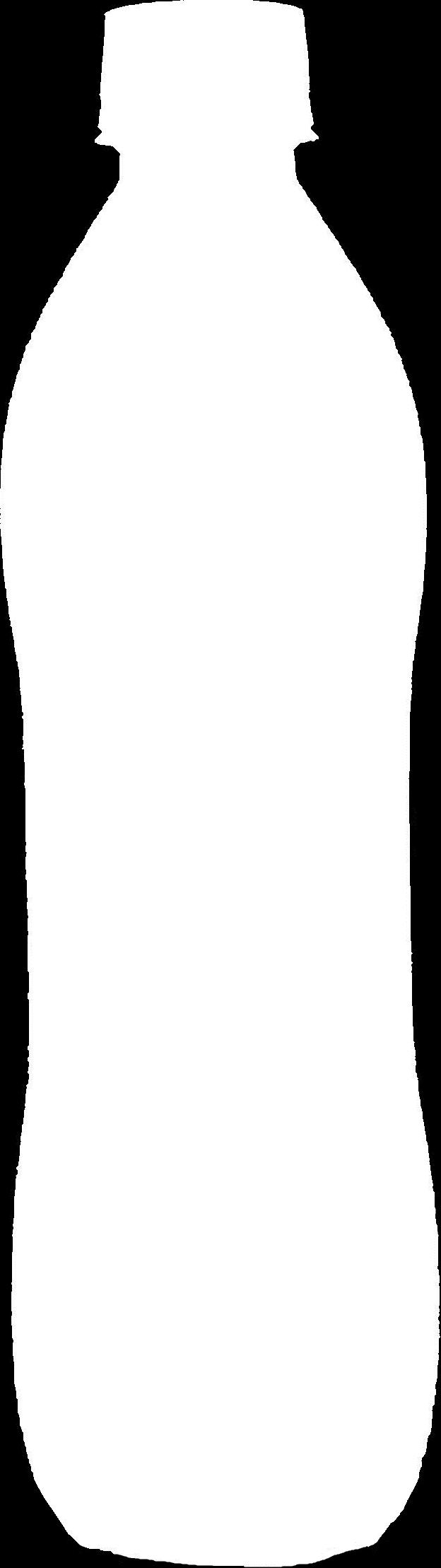

Supplement: Supplementary file 4 — Supplementary material [file mmc4.zip › Stimuli/Soda6.JPG]

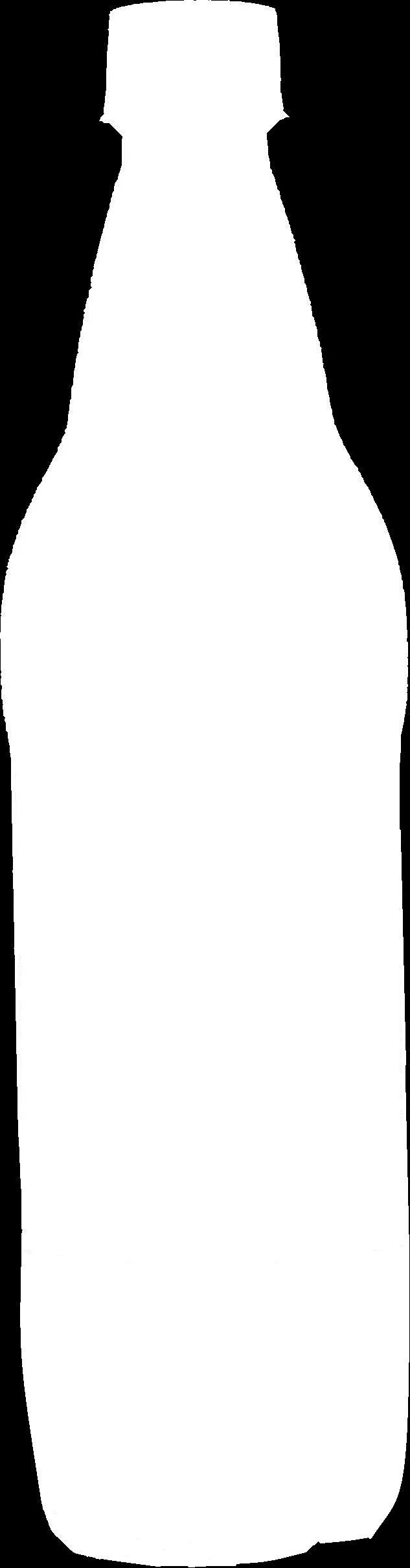

Supplement: Supplementary file 4 — Supplementary material [file mmc4.zip › Stimuli/Soda7.JPG]

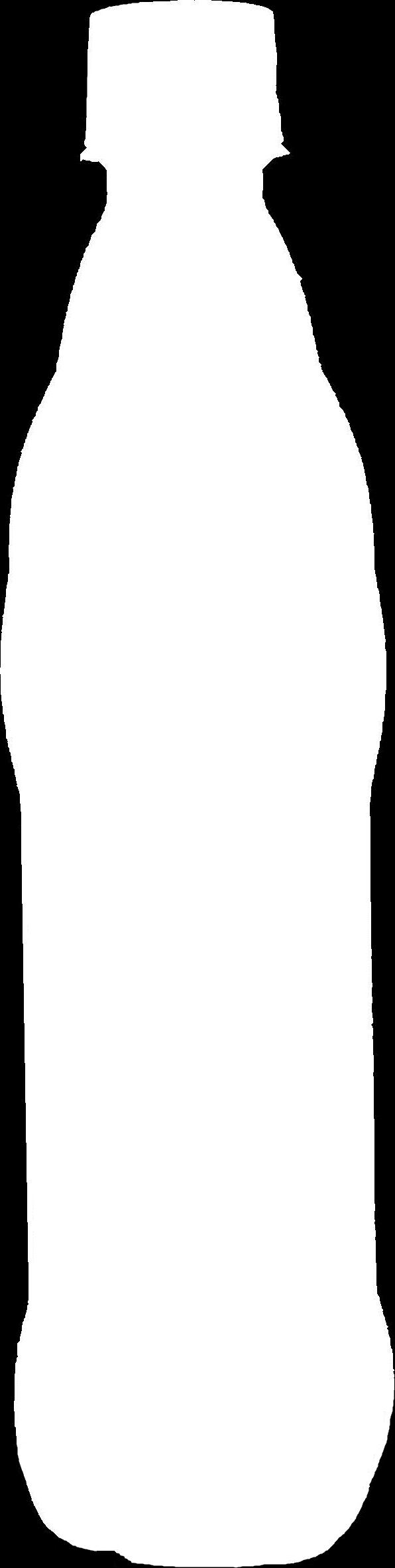

Supplement: Supplementary file 4 — Supplementary material [file mmc4.zip › Stimuli/Soda8.JPG]

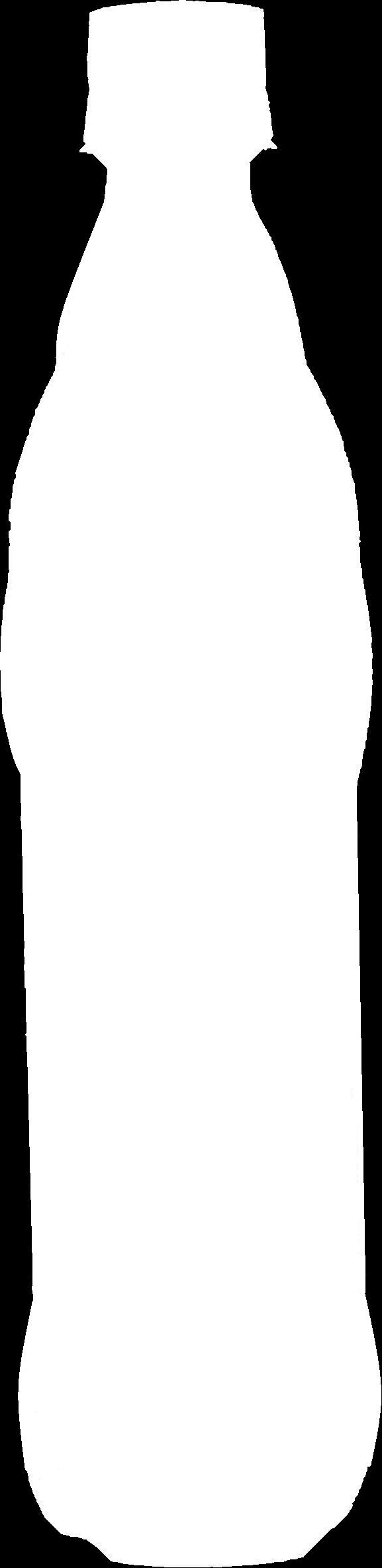

Supplement: Supplementary file 4 — Supplementary material [file mmc4.zip › Stimuli/Soda9.JPG]

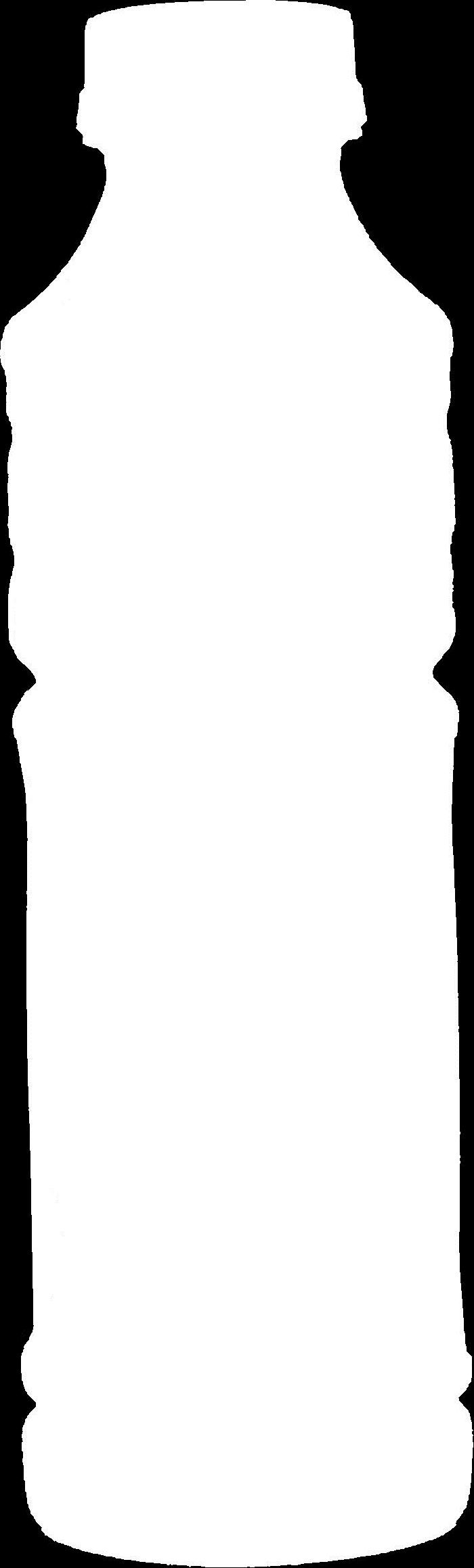

Supplement: Supplementary file 4 — Supplementary material [file mmc4.zip › Stimuli/SportsDrink1.JPG]

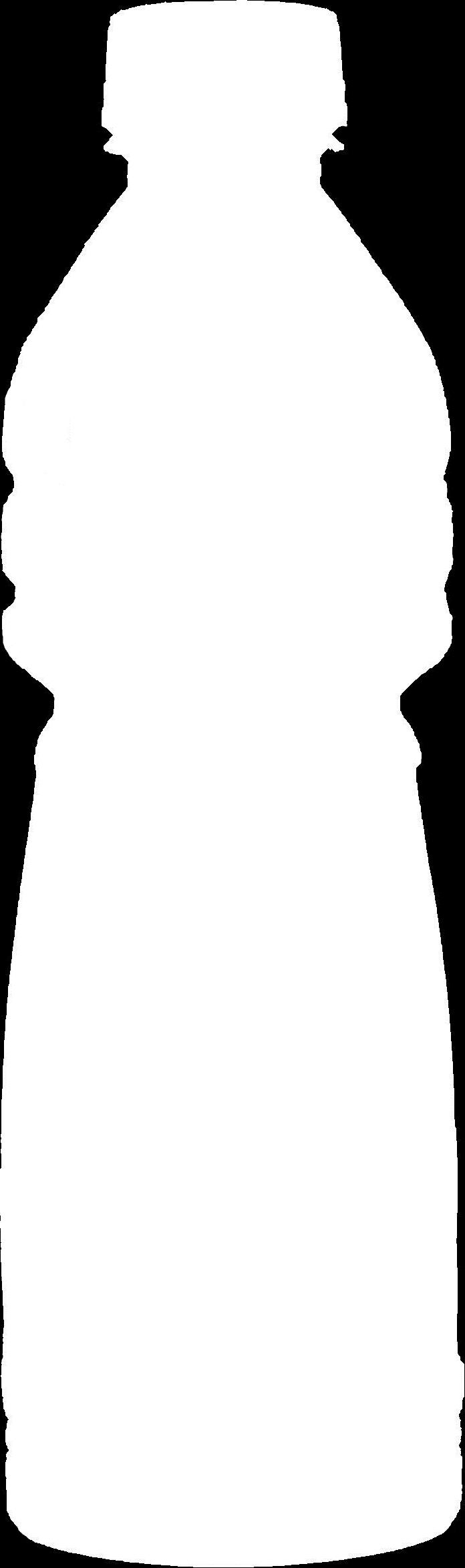

Supplement: Supplementary file 4 — Supplementary material [file mmc4.zip › Stimuli/SportsDrink2.JPG]

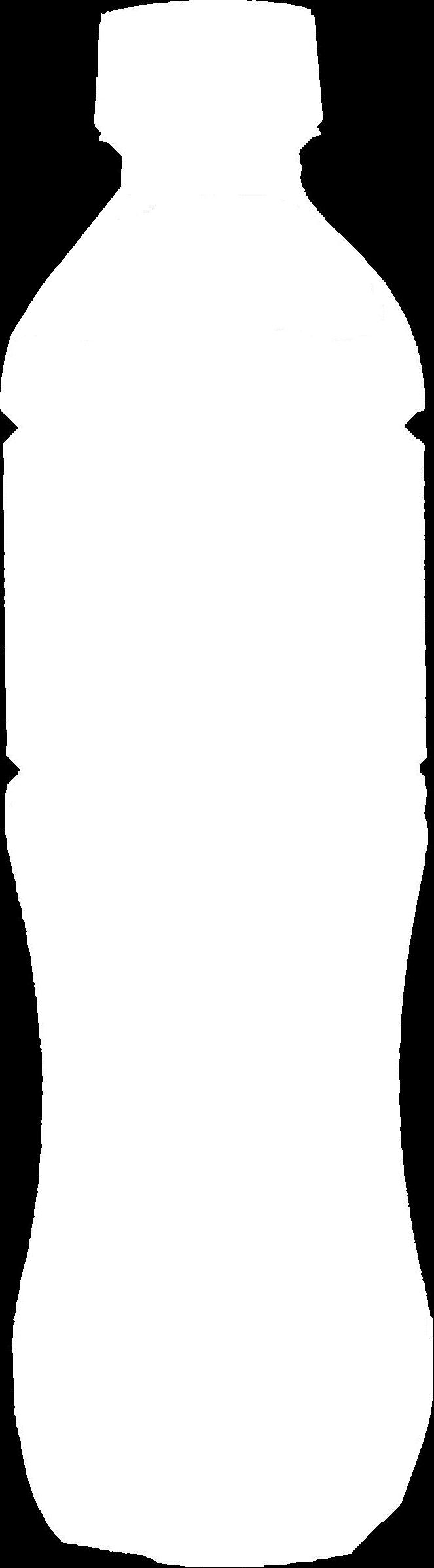

Supplement: Supplementary file 4 — Supplementary material [file mmc4.zip › Stimuli/SportsDrink3.JPG]

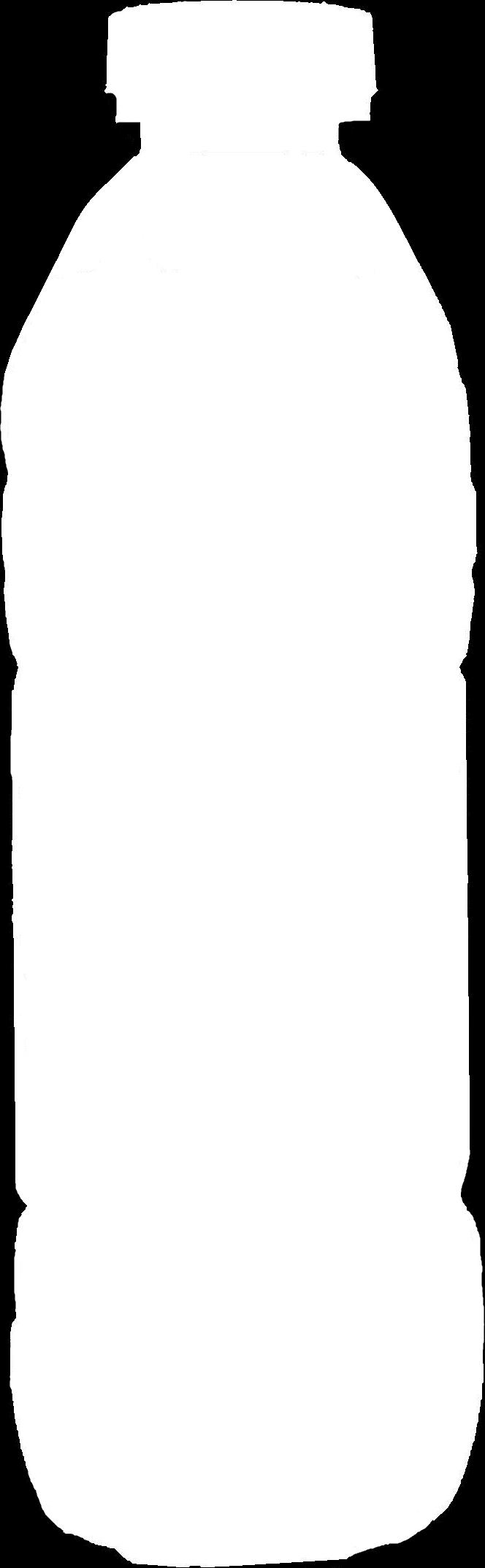

Supplement: Supplementary file 4 — Supplementary material [file mmc4.zip › Stimuli/Tea1.JPG]

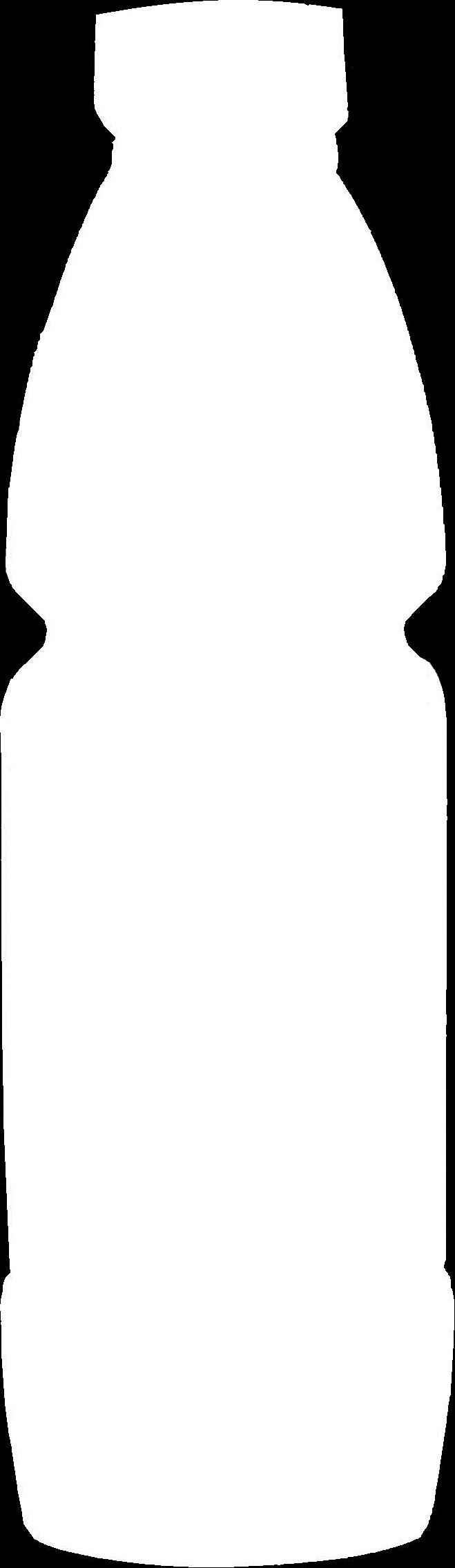

Supplement: Supplementary file 4 — Supplementary material [file mmc4.zip › Stimuli/Tea2.JPG]

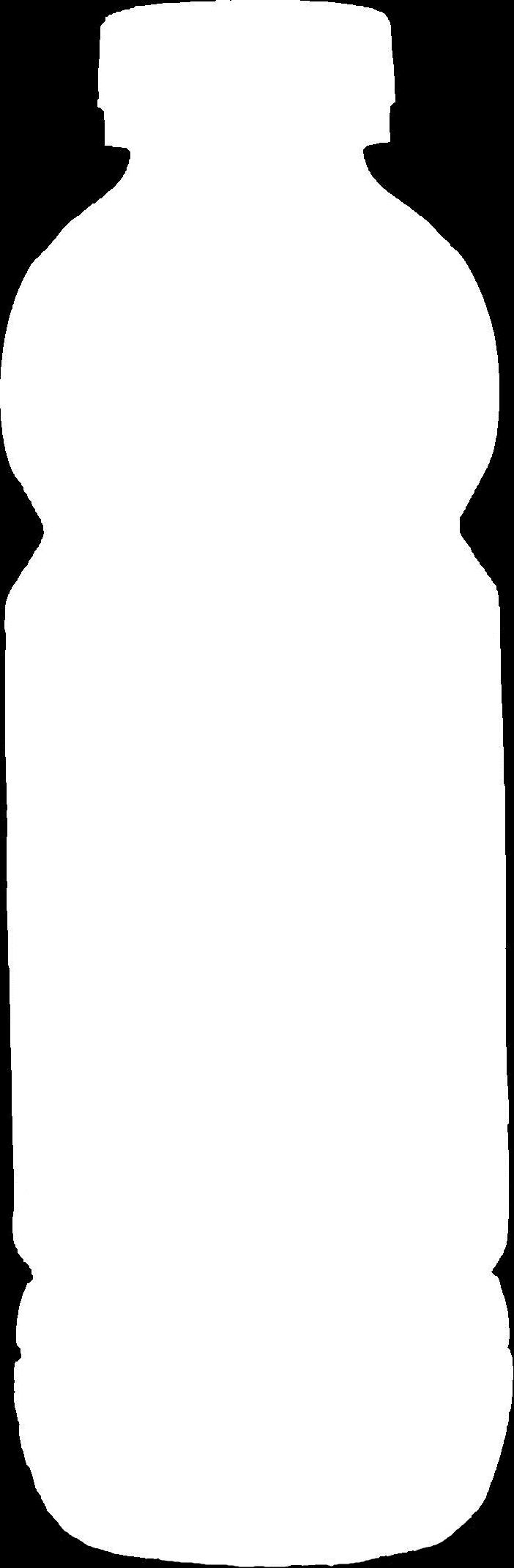

Supplement: Supplementary file 4 — Supplementary material [file mmc4.zip › Stimuli/Tea3.JPG]

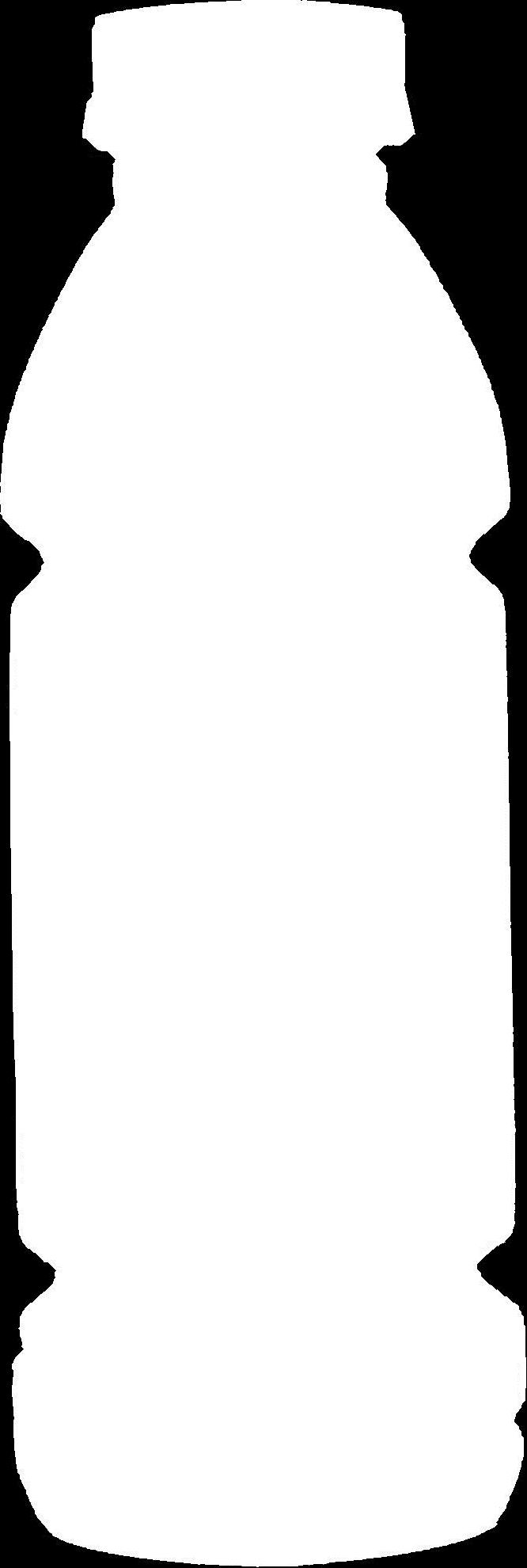

Supplement: Supplementary file 4 — Supplementary material [file mmc4.zip › Stimuli/Tea4.JPG]

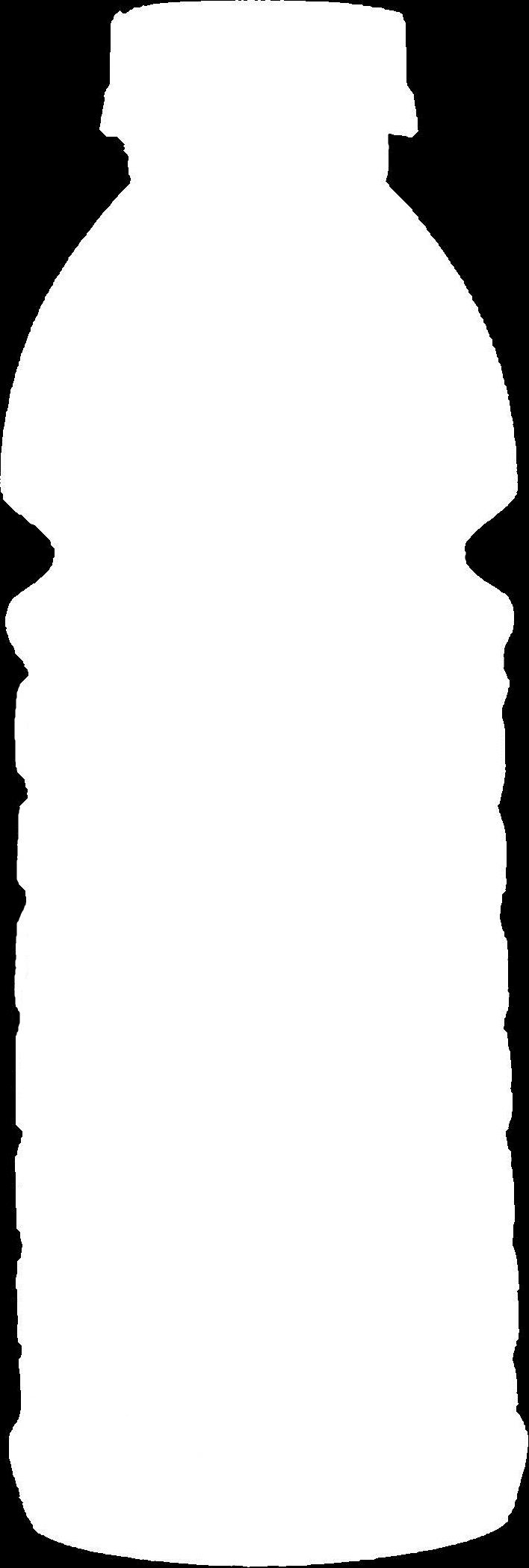

Supplement: Supplementary file 4 — Supplementary material [file mmc4.zip › Stimuli/Tea5.JPG]

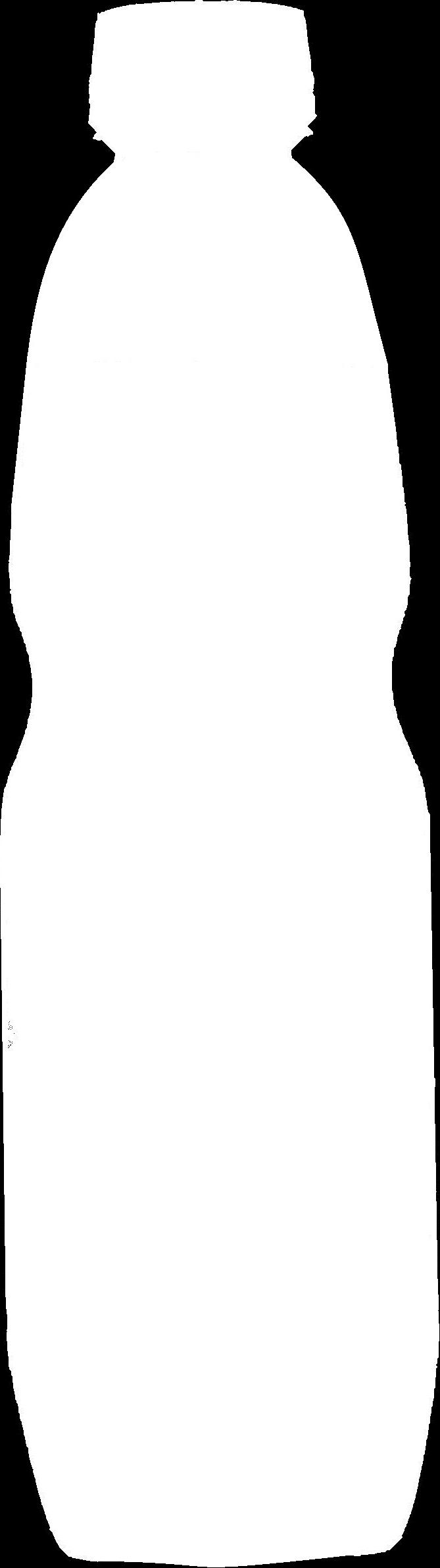

Supplement: Supplementary file 4 — Supplementary material [file mmc4.zip › Stimuli/Tea6.JPG]

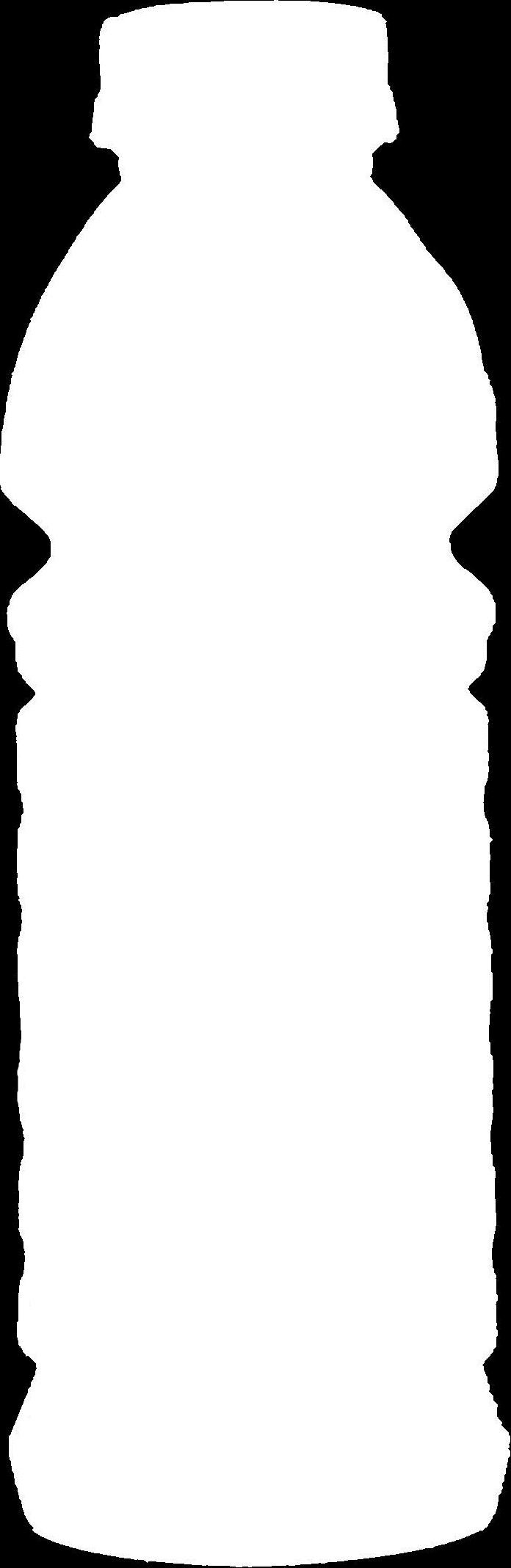

Supplement: Supplementary file 4 — Supplementary material [file mmc4.zip › Stimuli/Tea7.JPG]

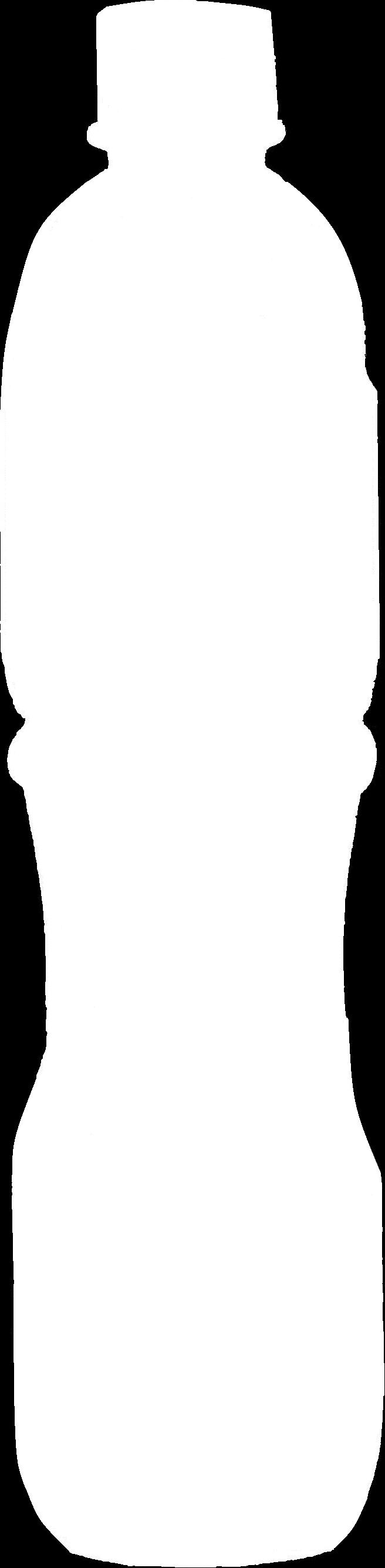

Supplement: Supplementary file 4 — Supplementary material [file mmc4.zip › Stimuli/Water1.JPG]

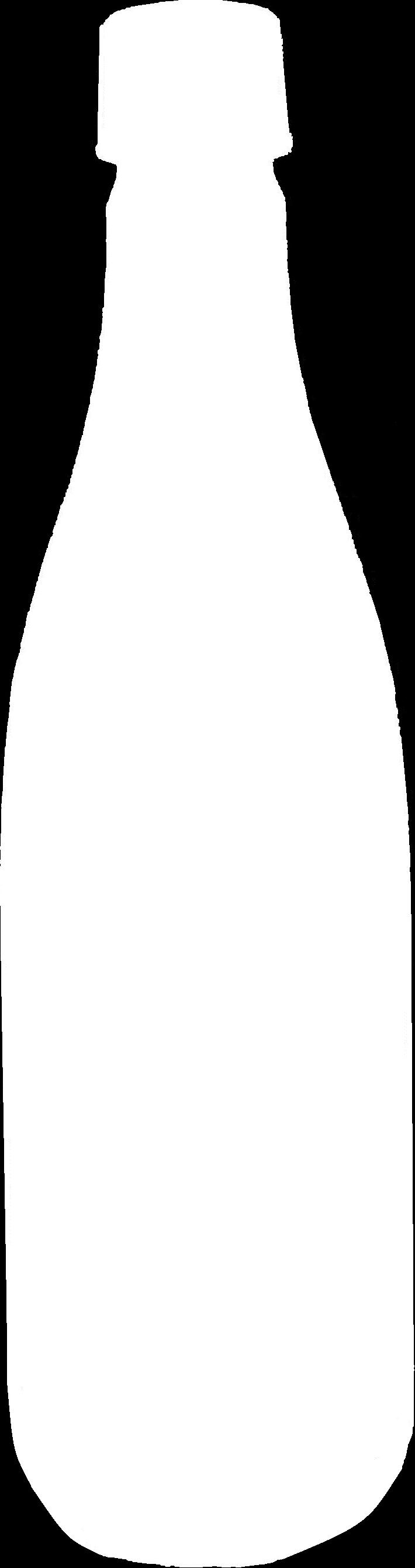

Supplement: Supplementary file 4 — Supplementary material [file mmc4.zip › Stimuli/Water2.JPG]

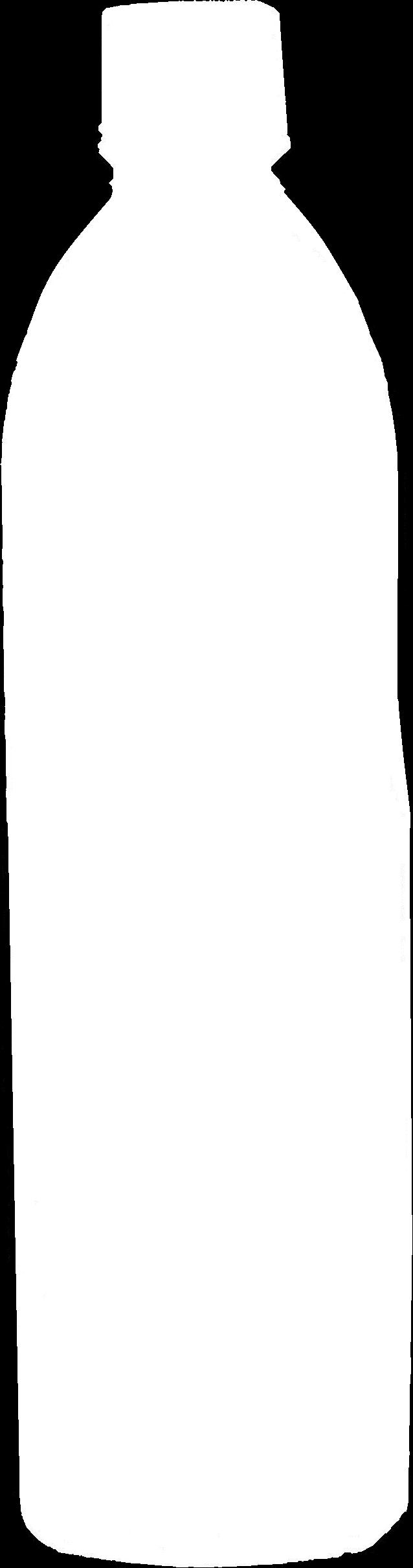

Supplement: Supplementary file 4 — Supplementary material [file mmc4.zip › Stimuli/Water3.JPG]

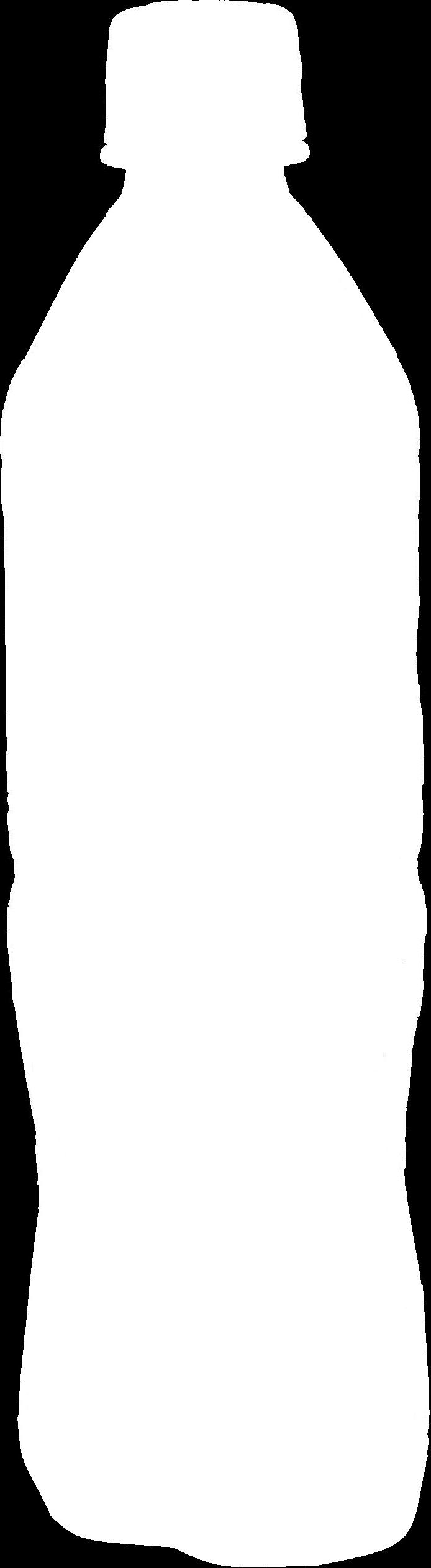

Supplement: Supplementary file 4 — Supplementary material [file mmc4.zip › Stimuli/Water4.JPG]

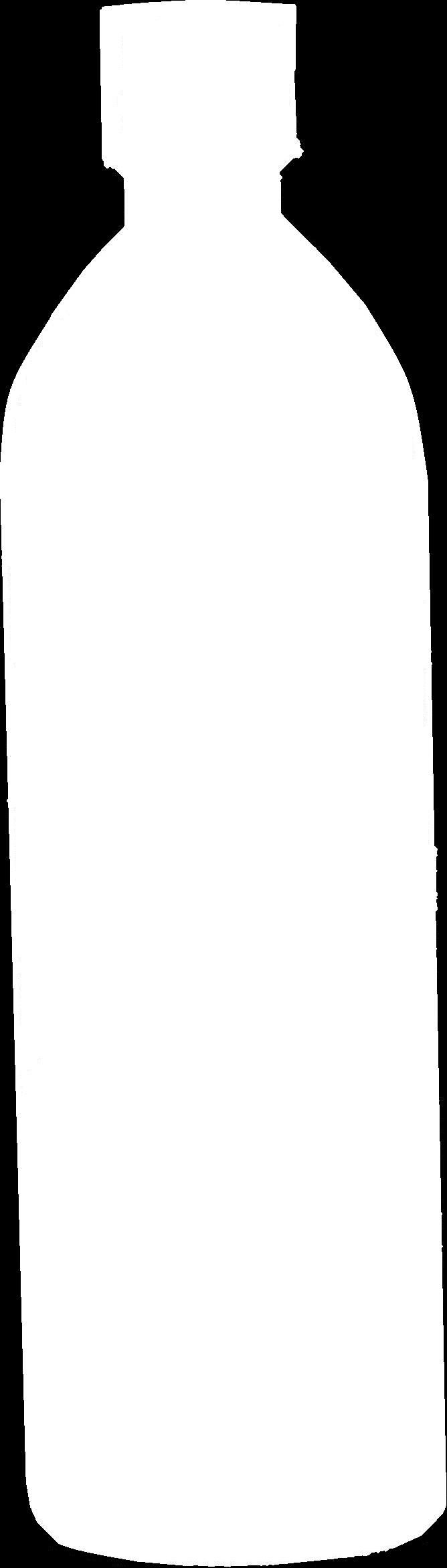

Supplement: Supplementary file 4 — Supplementary material [file mmc4.zip › Stimuli/Water5.JPG]

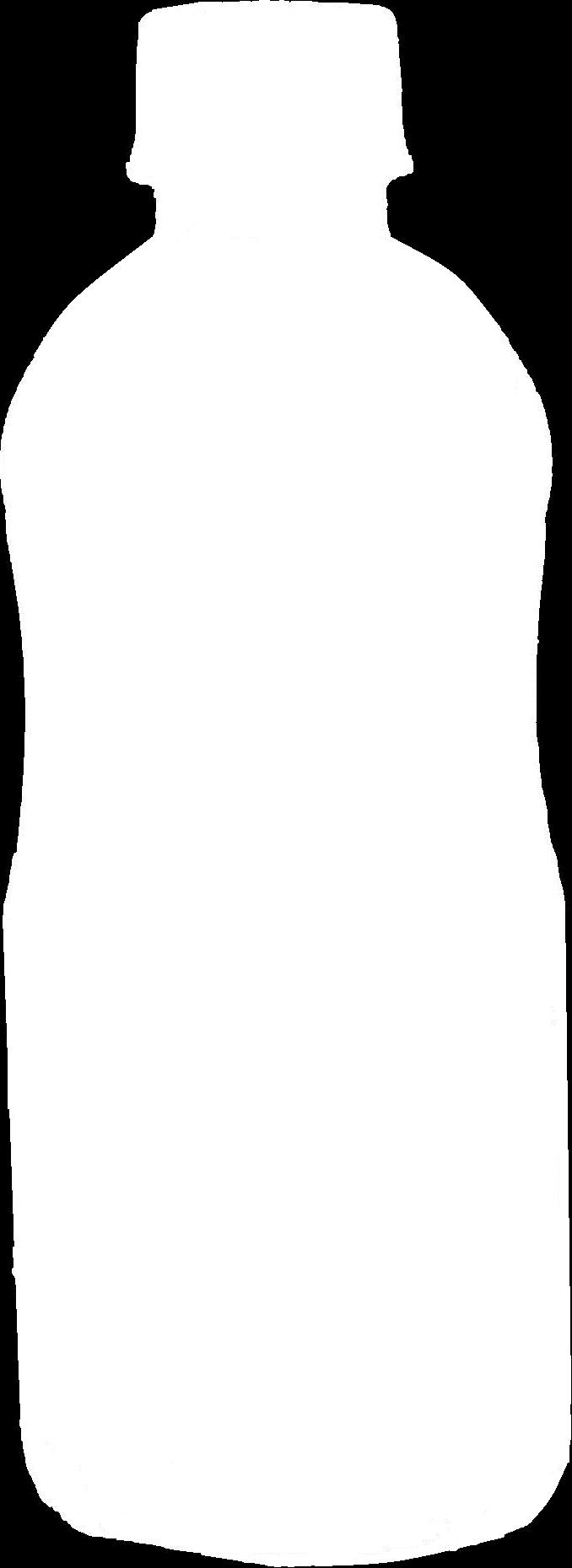

Supplement: Supplementary file 4 — Supplementary material [file mmc4.zip › Stimuli/Water6.JPG]

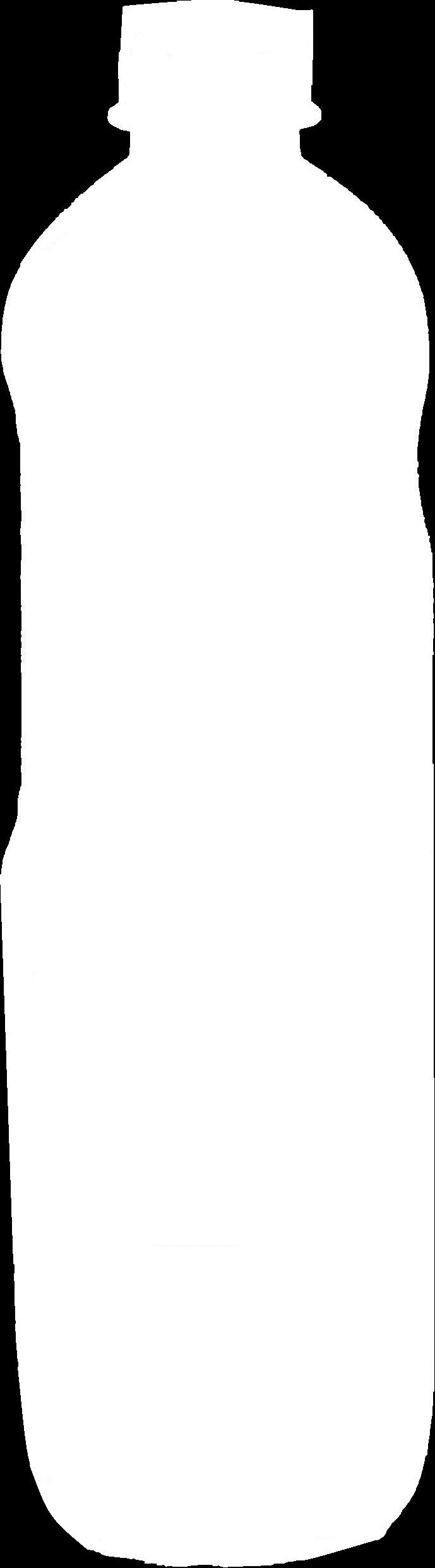

Supplement: Supplementary file 4 — Supplementary material [file mmc4.zip › Stimuli/Water7.JPG]

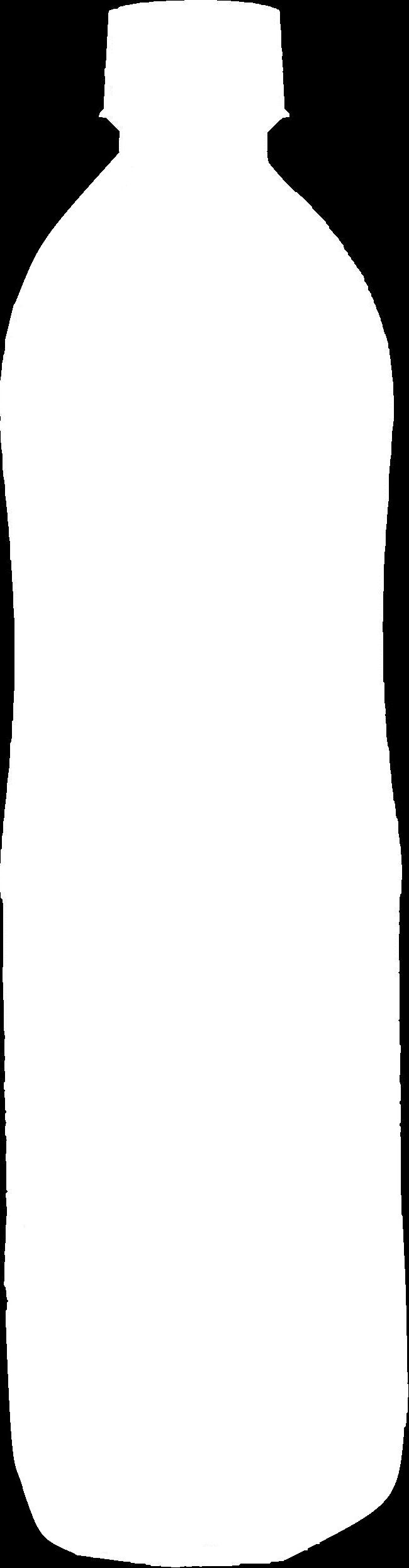

Supplement: Supplementary file 4 — Supplementary material [file mmc4.zip › Stimuli/Water8.JPG]
